# Computational Insights into the Mechanism of Lewis Acid‐Catalyzed Alkene‐Aldehyde Coupling

**DOI:** 10.1002/cplu.202400751

**Published:** 2025-02-11

**Authors:** Ricardo Meyrelles, Bogdan R. Brutiu, Boris Maryasin

**Affiliations:** ^1^ Institute of Organic Chemistry Faculty of Chemistry University of Vienna Währinger Straße 38 1090 Vienna Austria; ^2^ Institute of Theoretical Chemistry Faculty of Chemistry University of Vienna Währinger Straße 17 1090 Vienna Austria; ^3^ Vienna Doctoral School in Chemistry University of Vienna Währinger Straße 42 1090 Vienna Austria

**Keywords:** Cyclization, Redox neutral reactions, Hydride shifts, DFT, Reaction mechanisms

## Abstract

The Lewis acid‐catalyzed coupling of alkenes and aldehydes presents a modern, versatile synthetic alternative to classical carbonyl addition chemistry, offering exceptional regio‐ and stereoselectivity. In this work, we present a comprehensive computational investigation into the reaction mechanism of this transformation. Our findings confirm the occurrence of an enantioselective transannular [1,5]‐hydride shift step and demonstrate that the enantioselectivity of the reaction arises predominantly from steric clashes between functional groups in the cyclization step. Combining computational and experimental results, we establish that the Lewis acid catalyst facilitates the initial C−O coupling step between the alkene and the activated aldehyde. Investigations into systems with longer alkyl chains reveal that while they follow a similar mechanistic pathway, cyclization becomes kinetically hindered, preventing the reaction from proceeding. These insights illuminate the factors governing reaction outcomes and limitations, paving the way for future developments in this area.

## Graphical abstract

The reaction mechanism of an enantioselective Lewis acid‐catalyzed coupling of an alkene and an aldehyde was investigated using DFT methods. The study revealed two key steps in the transformation: cyclization and a hydride shift. Attempts with an alkene substrate bearing a longer alkyl chain failed to yield the expected product. Further computational analysis revealed that this experimental outcome is due to a hindered cyclization step

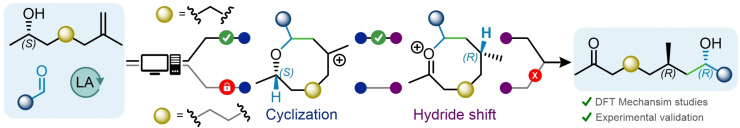

## Introduction

**Scheme 1:**
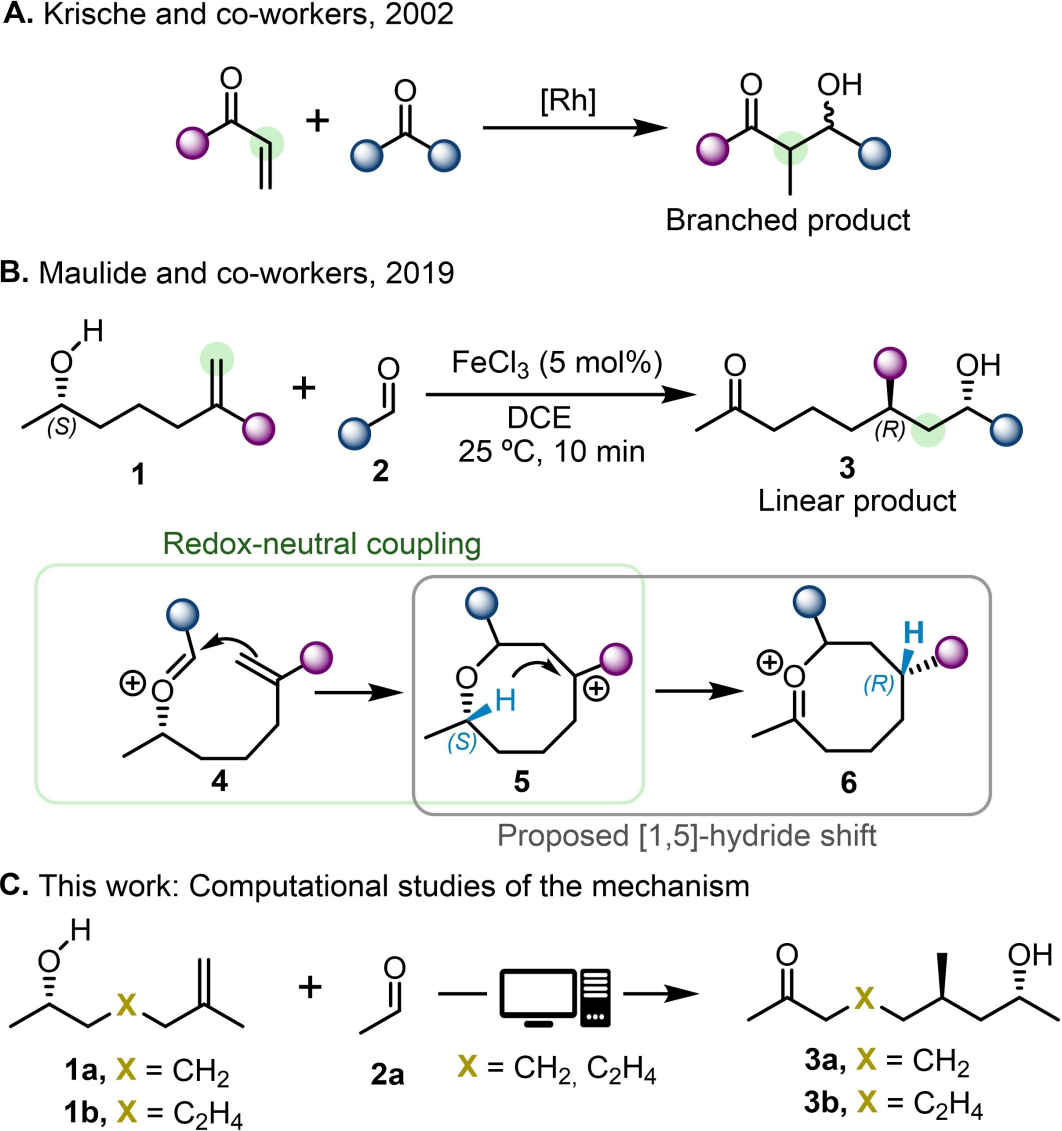
Reported coupling reactions by A) Krische and co‐workers; B) Maulide and co‐workers, along with proposed mechanistic steps. C) Coupling reaction investigated for alkene substrates with varying alkyl chain lengths through in‐depth *in silico* studies.

Over recent years, the coupling reactions of aldehydes and alkenes have been significantly developed. These reactions pose an efficient and highly atom‐economical process for the formation of carbon‐carbon bonds, relying on simple reactants that can often be obtained from readily available feedstocks. At the same time, their coupling products are considered highly valuable synthetic targets, particularly in natural product synthesis.[^1^, ^2^, ^3^, ^4^] The appeal of these transformations has inspired a variety of studies, with notable contributions from the research groups of Krische and Buchwald, which primarily demonstrated the formation of branched products (Scheme 1A).[^5^, ^6^, ^7^, ^8^, ^9^, ^10^, ^11^, ^12^, ^13^, ^14^] These transformations rely on the use of transition metal complexes to catalyze the coupling reaction. Recently, Maulide and co‐workers have reported the application of Lewis acid (LA) catalysis to selectively obtain linear products of the coupling of aldehydes and chiral alkenes (Scheme 1B).[^15^, ^16^] One key feature of this approach is the activation of the aldehyde by the LA catalyst, allowing a redox‐neutral carbon‐carbon coupling to occur from a linear intermediate, **4**. The reported transformation proposed a mechanism involving [1,5]‐hydride shift from a tertiary carbocation, **5** (Scheme 1B), to form an 8‐membered oxocarbenium intermediate, **6**. However, no computational studies have yet been published to confirm or challenge this hypothesized pathway.^[17]^ Hydride shifts are a class of rearrangement reactions involving the migration of a hydrogen atom within a carbocation.[^18^, ^19^] These transformations are a common feature of carbocation chemistry and are particularly important in the biological pathways by which steroids and related substances are synthesized.^[20]^ However, such intramolecular rearrangements occur extremely rapidly, making them challenging to study experimentally. In contrast, computational methods are particularly well‐suited for exploring the underlying mechanisms of such transformations.

Notably, the proposed transannular [1,5]‐hydride shift is synthetically appealing as it allows a unique metal‐free C−H functionalization event.[^21^, ^22^] Experimentally, this reaction demonstrated remarkable enantioselectivity, exclusively producing *(R,R)‐*configured products when employing an *(S)*‐configured alkene. The study of the underlying mechanism in greater detail is thus highly important, as it could provide insights into controlling this reaction and enabling further applications. The key questions addressed in our in‐depth *in silico* studies are whether we can confirm if this process involves a hydride shift step and if the reaction could also operate in the context of larger ring sizes, arising from longer alkyl chains in the alkene substrate (Scheme 1C).

## Results and Discussion

The reaction profile leading to the cyclic oxocarbenium intermediate equivalent to **6** (Scheme 1B) was computationally investigated using density functional theory DFT: PBE0‐D3(BJ), SMD(DCM)/def2‐TZVP//PBE0‐D3(BJ),SMD(DCM)/def2‐SVP.[^23^, ^24^, ^25^, ^26^, ^27^, ^28^] The computational model was constructed by adapting the reported experimental conditions.^[16]^ Dichloromethane was considered for the implicit solvation model instead of the experimentally used dichloroethane, and BF_3_ was employed as a substitute for the experimentally used Lewis acid, FeCl_3_. It was shown that BF_3_ can promote the coupling reaction, although with lower efficiency.^[15]^ Additionally, an explicit dichloromethane molecule was included in the calculations to aid in the stabilization of the charged species.

**Scheme 2:**
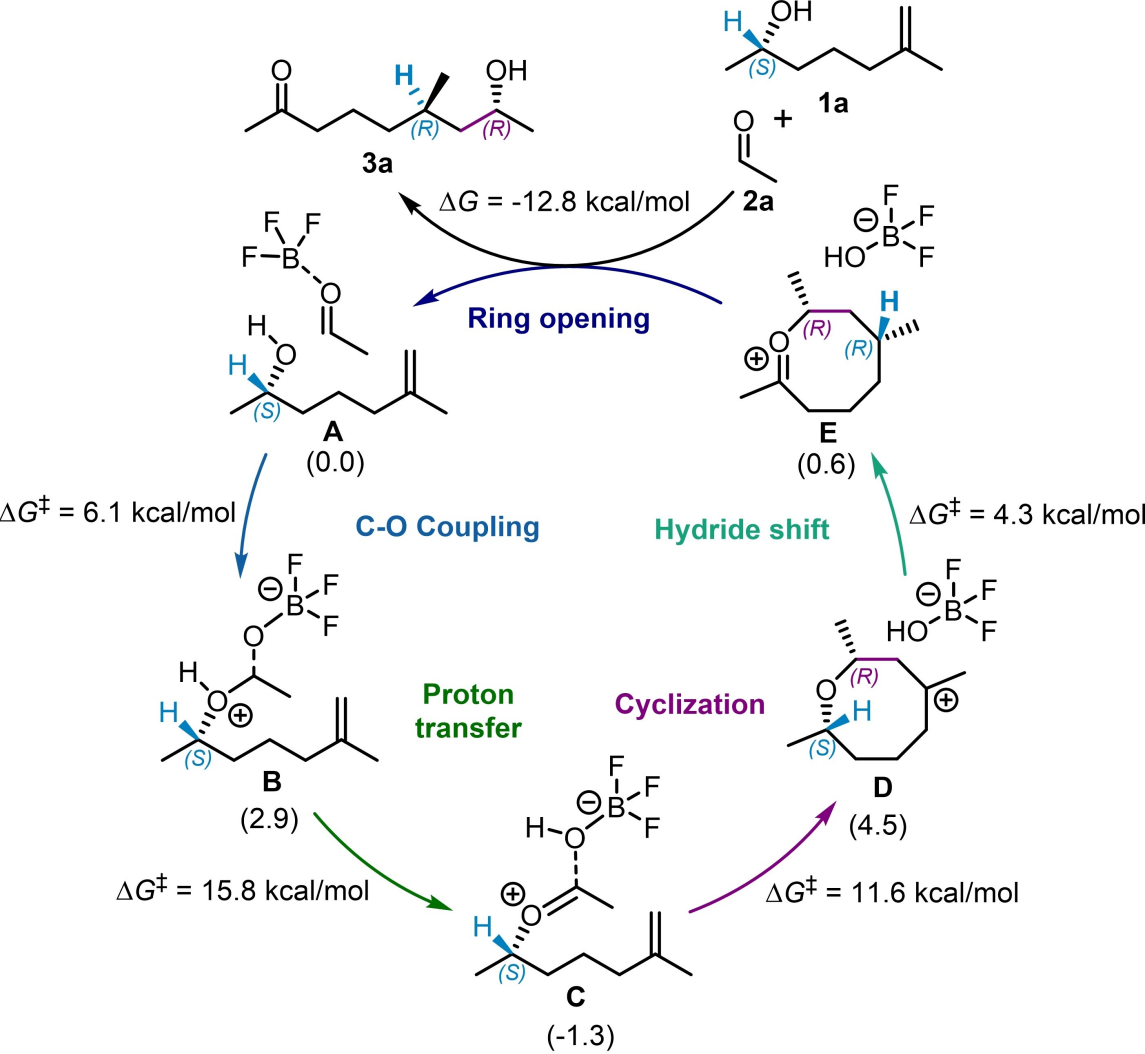
Computed catalytic cycle. The Gibbs free energy values in parentheses are in reference to reactant complex **A** (0.0 kcal/mol), and the Gibbs free activation energy barriers (Δ*G* ^≠^) are presented for each individual step.

The computed catalytic cycle, illustrated in Scheme 2, begins with the formation of the reactant complex **A**. This complex consists of the *(S)‐*enantiomer of the chiral alkene **1 a** (as depicted in Scheme 1) and an adduct of the formaldehyde **2 a** with the Lewis acid catalyst BF_3_. From the reactant complex **A**, the activated aldehyde undergoes nucleophilic attack by the hydroxy group of the chiral alkene, leading to the formation of intermediate **B**. This step is slightly endergonic (Δ*G*(**A**→**B**)=2.9 kcal/mol) but features a very low activation barrier (Δ*G* ^≠^(**A**→**B**)=6.1 kcal/mol), making it both fast and reversible. The formation of transient intermediate **B** facilitates an exergonic proton transfer event (Δ*G*(**B**→**C**)=−4.2 kcal/mol), resulting in intermediate **C**. This intermediate **C** can be described as an adduct of an oxocarbenium ion and BF_3_OH^−^. This proton transfer step proceeds via a four‐membered ring transition state, resulting in a relatively high activation energy barrier (Δ*G* ^≠^(**B**→**C**)=15.8 kcal/mol). The subsequent step involves cyclization, which occurs via the formation of a carbon‐carbon bond with the terminal alkene (**C**→**D**). During this process, the O−C bond between the oxocarbenium ion and the Lewis acid adduct is cleaved, releasing BF_3_OH^−^ Simultaneously, a new C−C bond is established, yielding an eight‐membered tertiary carbocation intermediate **D** with a relatively low kinetic barrier, Δ*G* ^≠^(**C**→**D**)=11.6 kcal/mol. Notably, this step also leads to the formation of a new chiral center, highlighted in purple in Scheme 2. Finally, intermediate **D** undergoes a transannular [1,5]‐hydride shift, resulting in the formation of the 8‐membered oxocarbenium intermediate **E**. This hydride shift facilitates a chirality transfer, as the *(S)‐*configured carbon initially bound to oxygen loses its tetrahedral geometry, while the newly formed tertiary carbocation adopts an *(R)‐*configuration. Although the formation of **D** is endergonic (Δ*G*(**C**→**D**)=5.8 kcal/mol), the subsequent [1,5]‐hydride shift is kinetically favored over the reversal of the cyclization step, facilitating the transformation of **D** into **E** (Δ*G* ^≠^(**D**→**E**)=4.3 kcal/mol compared to Δ*G* ^≠^(**D**→**C**)=5.8 kcal/mol). Finally, a ring‐opening event involving the incorporation of an OH^−^ fragment regenerates the Lewis acid catalyst BF_3_. The conversion of the reactants, **1 a** and **2 a**, into the product, **3 a**, is an exergonic process with Δ*G*(**1 a**+**2 a**→**3 a**)=−12.8 kcal/mol.

Using the energetic span model,^[29]^ the apparent activation barrier for this process is determined to be 18.7 kcal/mol, corresponding to the free energy difference between the rate‐determining intermediate **A** and the rate‐determining transition state **TS_BC_**. This barrier aligns well with the reaction conditions and remains unaffected by the cyclization or the [1,5]‐hydride shift steps.

**Figure 1:**
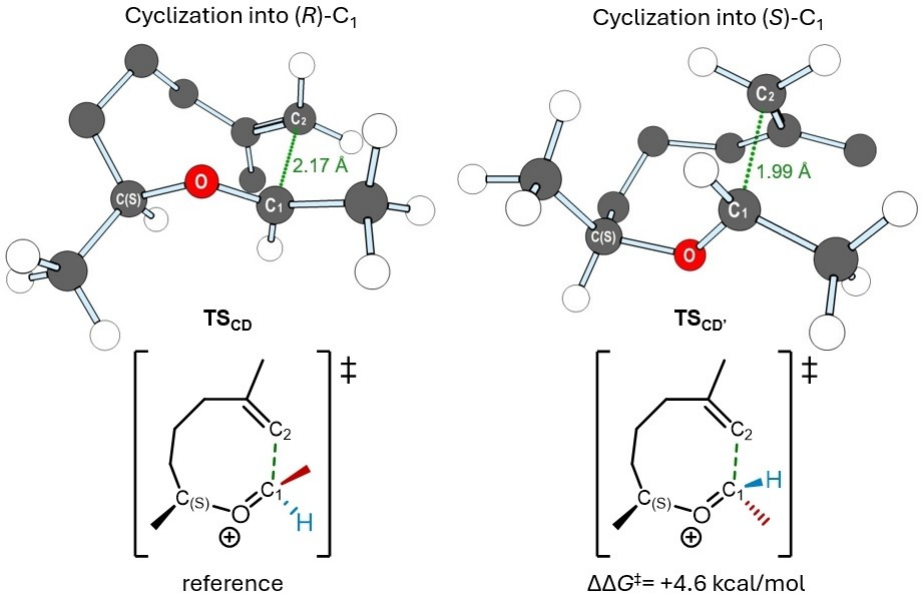
Representation of the optimized structures of the transition states for the cyclization step leading to *(R)*‐configured (left, **TS_CD_**) and *(S)‐*configured (right, **TS_CD’_**) intermediates **D** and **D’** respectively, corresponding to the C_1_ chiral center. Selected hydrogen atoms are hidden for simplicity. The Gibbs free energy gap between the respective barriers is shown, taking the barrier of **TS_CD_** as a reference (ΔΔ*G* ^≠^=0.0 kcal/mol).

As previously mentioned, the cyclization step **C**→**D** (Scheme 2, purple) introduces a new chiral center into the system. Consequently, this step is responsible for the potential enantioselectivity. To confirm the observed selectivity favoring the formation of an *(R)‐*configured center in intermediate **D**, the transition state for cyclization leading to an *(S)‐*configured intermediate, **TS_CD_’**, was also computed. A three‐dimensional representation of the geometry of the most stable conformations for the two competing transition states, leading to *(R)‐* or *(S)‐*configured products, is presented in Figure 1. The cyclization transition state leading to an *(S)‐*configuration at C_1_ (**TS_CD’_**) is 4.6 kcal/mol less stable than **TS_CD_**. The reduced stability of **TS_CD’_** is evident from the shorter C_1_−C_2_ bond distance (1.99 Å), which is 0.19 Å less than the corresponding distance in **TS_CD_** (2.17 Å). This indicates that the structure of **TS_CD’_** requires a greater distortion of the equilibrium geometry **C**, forcing the carbons closer together and suggesting a late transition state. Notably, in the most favorable conformations of both transition states, the methyl substituent on carbon **C_1_** adopts the equatorial position. However, the methyl substituent on carbon C(S) adopts the equatorial position only in **TS_CD_**. In **TS_CD’_**, this group is forced into an axial position, bringing it closer to the alkene group (C_2_) and increasing steric repulsion. This results in heightened transannular strain within the ring structure, ultimately destabilizing the transition state and raising the kinetic barrier for cyclization (see ESI for details). Consequently, the kinetic preference for **TS_CD_** drives the complete enantioselectivity observed in the cyclization step.

**Figure 2:**
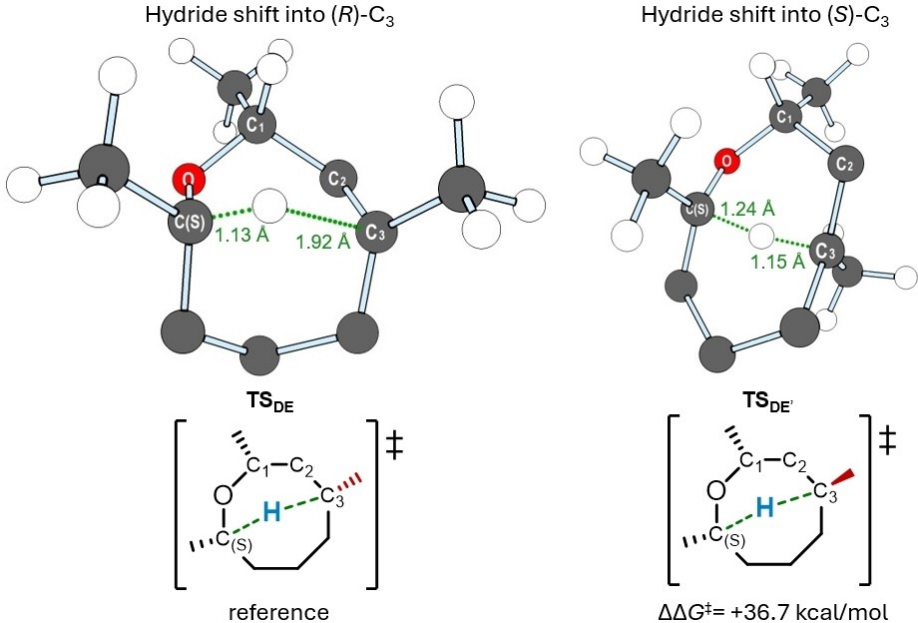
The optimized structures of the transition states for the [1,5]‐hydride shift step leading to *(R)*‐configured (left, **TS_DE_**) and *(S)‐*configured (right, **TS_DE’_**) intermediates **E** and **E’** respectively, corresponding to the C_3_ chiral center. Selected hydrogen atoms are hidden for simplicity. The Gibbs free energy gap between the respective barriers is shown, taking the barrier of **TS_DE_** as a reference (ΔΔ*G* ^≠^=0.0 kcal/mol).

The three‐dimensional structure of the transition state for the [1,5]‐hydride shift **TS_DE_** (the step is highlighted in green within the catalytic cycle in Scheme 2) is depicted in Figure 2, left. This critical step enables a chirality transfer from the *(S)‐*configured carbon C(S) to C_3_, which adopts an *(R)‐*configuration following the formation of the C_3_−H bond. This hydride shift results in a bridged bicyclic transition state structure, where the C(S)−H bond is cleaved at a shorter length (1.13 Å) than the C_3_−H bond is formed (1.92 Å), indicative of an early transition state. As an alternative pathway, the hydrogen atom could migrate to the opposite face of the carbocation, leading to the formation of an *(S)‐*configured center. The transition state for this alternative [1,5]‐hydride shift, referred to as **TS_DE’_**, was also computed (Figure 2, right). In contrast to **TS_DE_**, **TS_DE’_** represents a late transition state, requiring the C(S)−H bond to stretch to 1.24 Å, with the C_3_−H bond forming only when the nuclei are 1.15 Å apart. Furthermore, this hydride shift involves a significant contraction of the 8‐membered ring, resulting in the formation of a bridged bicyclic structure with a C(S)−H−C_3_ angle of 169° (compared to 123° in **TS_DE_**). Consequently, **TS_DE’_** exhibits considerable thermodynamic instability relative to **TS_DE_**, being 36.7 kcal/mol less stable. This renders **TS_DE’_** kinetically prohibitive, thereby supporting the proposed enantioselective pathway leading to the formation of the linear coupling product.

**Scheme 3:**
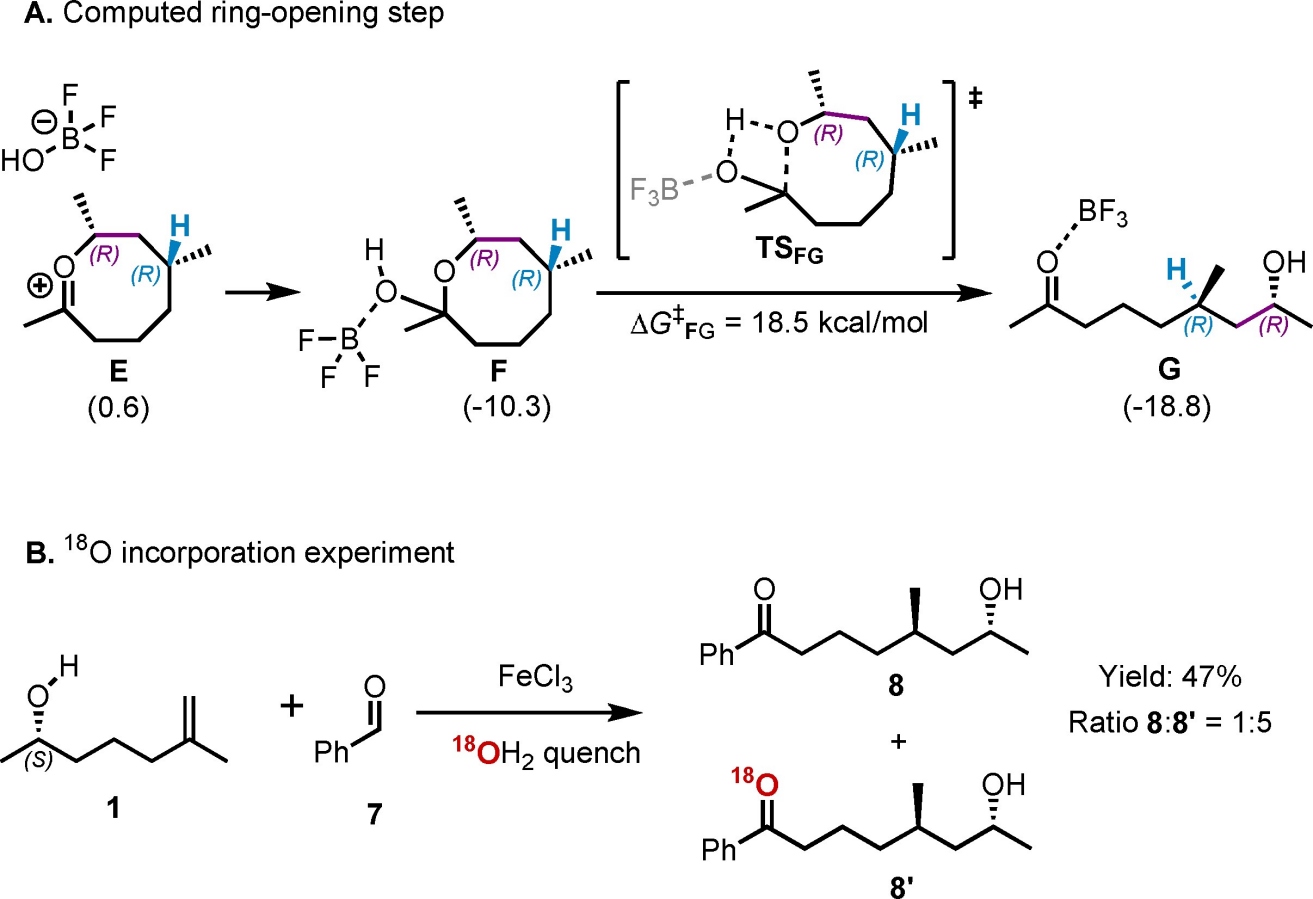
A) The computational analysis of the alternative ring‐opening event via catalyst rebound. The values in parentheses are relative Gibbs free energy values in kcal/mol, in reference to reactant complex **A**. B) The labeling experiment with heavy oxygen water.

In the next stage, we sought to understand the mechanism behind the opening of the 8‐membered ring, which leads to the experimentally observed final product. Initially, we hypothesized that the catalyst might facilitate this final step. As illustrated in Scheme 3A, the complex **F** can form through the rebound of the LA catalyst adduct, BF_3_OH^−^, with intermediate **E** in an exergonic process (Δ*G*(**E**→**F**)=−10.9 kcal/mol). Subsequently, **F** undergoes ring opening via a 4‐membered ring transition state, **TS_FG_**, with a moderate kinetic barrier of 18.5 kcal/mol. The formation of the product complex **G** is exergonic (Δ*G*(**F**→**G**)=−8.5 kcal/mol), suggesting that such a mechanism for the regeneration of the catalyst is plausible.

To validate these findings, we performed a labeling mechanistic experiment using heavy oxygen water (H₂^1^⁸O), as shown in Scheme 3B. The reaction was performed with alkene **1 a** and phenylacetaldehyde, **7**. The product of the coupling reaction was obtained in 47 % isolated yield, revealing a ratio between the expected product, **8**, and the ^18^O labeled product, **8’**, of 1 : 5. These results strongly suggest that the ring opening step occurs mainly without catalyst assistance. Therefore, although a ring‐opening event, equivalent to the computed step **F**→**G** (Scheme 3A), is kinetically allowed, the LA catalyst can be regenerated early in the catalytic cycle. As a result, the LA adduct with hydroxide formed after the initial nucleophilic attack by the alcohol is able to regenerate the LA, and the reaction proceeds towards the formation of a stable oxocarbenium intermediate (**6** in Scheme 1B). This intermediate then remains in solution and only undergoes ring opening through hydrolysis during the quenching of the reaction. Thus, we can manifest that the Lewis acid catalyst plays a role mainly in the activation of the aldehyde during the initial coupling step.

With strong computational evidence of the occurrence of a transannular [1,5]‐hydride shift step within the reaction mechanism, the final question we addressed was whether the coupling reaction could proceed with a chain length extended by one additional carbon atom in the alkene. In particular, an increase in the length of the alkyl chain would result in the formation of 9‐membered intermediates instead of 8‐membered, as computed for the reported reaction (Scheme 2). This ring expansion could impact the feasibility of the key [1,5]‐hydride shift step, possibly due to an increase in the transannular strain.^[30]^

**Scheme 4:**
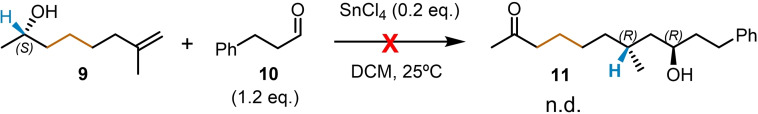
The experimental attempt at extending the carbon chain by one additional carbon atom (highlighted in orange) in the coupling reaction under investigation.

At first, a test reaction was attempted experimentally, following the reported protocol, with tin chloride as an LA catalyst (Scheme 4). From this reaction of aldehyde **10** and the chiral alkene **9**, containing an additional carbon in the alkyl chain (highlighted in orange), no coupling product, **11**, was detected. To better understand which factors inhibited the coupling reaction, we conducted further calculations.

**Scheme 5:**
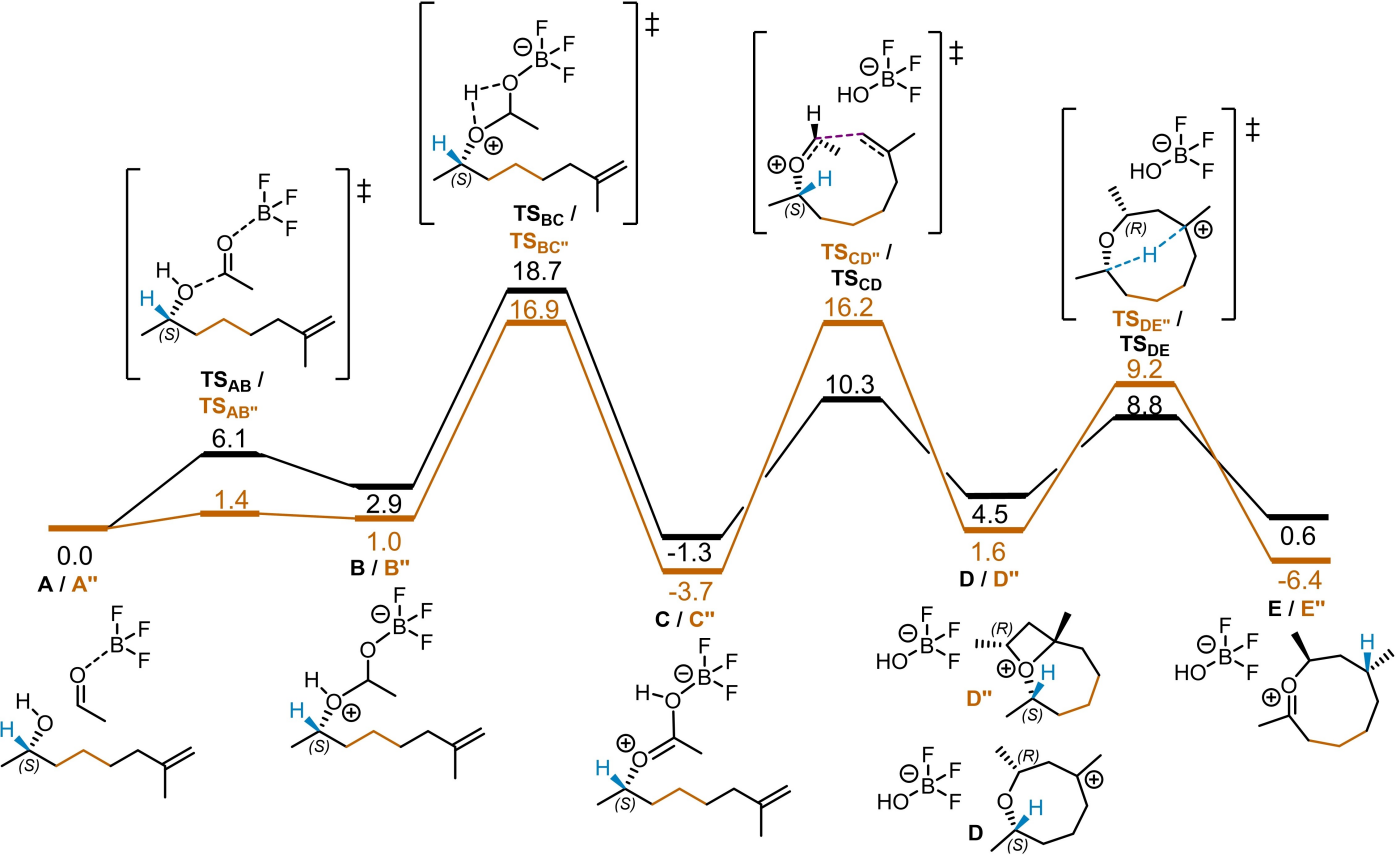
The computed Gibbs free energy (Δ*G*_298_) profiles for the initially investigated **1 a**→**3 a** reaction (black) and its longer chain analog (orange) in comparison. The reaction pathways are truncated for clarity, starting from the reactant (**A** and **A’’**) to the final product complexes (**E** and **E’’**). Reactant complexes **A** and **A’’** are taken as reference (0.0 kcal/mol).

Thus, we studied the equivalent mechanistic pathway for the experimentally failed reaction of an alkene with an additional carbon in the alkyl chain. Understanding which step prevents the success of this reaction is crucial. The proposed mechanistic pathway for this reaction is illustrated in Scheme 5 (highlighted in orange), alongside the corresponding steps from the mechanism of the initially studied shorter‐chain reaction (as shown in Scheme 2 for the catalytic cycle), presented in black in Scheme 5. For computational expediency, the phenylethyl group of **10** was replaced by methyl.

The mechanistic profile obtained for the reaction of formaldehyde with an alkene containing an additional carbon in the chain mirrors the same elemental steps as the previously discussed mechanism (with the catalytic cycle shown in Scheme 2). However, notable differences can be observed in the relative thermodynamic stabilities of the reaction intermediates and transition states. The profile for the extended alkyl chain (shown in orange) exhibits a lower activation barrier for the first two steps (**A’’**→**B’’** and **B’’**→**C’’**). These involve the nucleophilic attack of the alcohol on the activated aldehyde, followed by a subsequent proton transfer to form the oxocarbenium intermediate **C’’**. The longer alkyl chain likely reduces the steric repulsion between the terminal alkene and the activated aldehyde fragment, resulting in enhanced stability of these structures compared to the profile in black (with one less carbon in the alkyl chain). From **C’’**, the cyclization into a 9‐membered ring becomes the rate‐determining step (Δ*G* ^≠^(**C’’**→**D’’**)=19.9 kcal/mol). The formation of **D’’** is endergonic (Δ*G*(**C’’**→**D’’**)=5.3 kcal/mol), and this intermediate contains a 4‐membered oxocarbenium ring, formed through a C−O interaction that helps stabilize the cationic charge. This interaction is absent in **D** and likely results from the increased flexibility of the 9‐membered ring in **D’’**. The subsequent [1,5]‐hydride shift presents a higher activation barrier than the equivalent step in the black profile (Δ*G* ^≠^(**D’’**→**E’’**)=7.6 kcal/mol, compared to Δ*G* ^≠^(**D**→**E**)=4.3 kcal/mol). This can be attributed to the necessity of breaking the C−O interaction in the oxocarbenium intermediate **D’’**. The formation of the 9‐membered oxocarbenium intermediate **E’’** is exergonic (Δ*G*(**D’’**→**E’’**)=−8.0 kcal/mol). The thermodynamic stability of this structure is likely due to the low steric repulsion between the substituent groups in the flexible ring. These results demonstrate that extending the alkyl chain in the alkene reagent increases the flexibility of the intermediates, which facilitates the intramolecular stabilization of the cationic charge. Consequently, with more stable intermediates, the activation barriers for the cyclization and [1,5]‐hydride shift steps are elevated. This kinetic hindrance reduces the competitiveness of the pathway leading to the expected linear alkene products, making it more susceptible to alternative side reactions, such as intermolecular proton transfers that can produce many unidentified products. Therefore, the feasibility of the [1,5]‐hydride shift in ring structures is heavily dependent on the lower stability of the preceding intermediates.

## Conclusions

In this study, we have conducted a comprehensive computational investigation of the Lewis acid‐catalyzed coupling of alkenes and aldehydes discovered by Maulide and co‐workers. Our calculations not only elucidate the experimentally observed enantioselectivity but also provide a detailed understanding of all key reaction steps, including chirality transfer and the potential for catalyst rebound. These computational insights are fully supported by experimental evidence. Additionally, we have demonstrated why extending the alkyl chain by just one carbon atom renders the reaction infeasible. Specifically, we show that while the carbon chain elongation stabilizes the reaction intermediates, it inadvertently increases the energetic span, disrupting the catalytic cycle. The key challenge for the longer‐chain reaction is the cyclization step, where the higher activation barrier poses a significant obstacle. Therefore, future efforts to expand the scope of this reaction should primarily focus on strategies to lower the cyclization barrier, enabling the reaction to accommodate longer chains.

## Experimental Section

**Computational details**: The conformational space of all molecules has been initially searched using meta‐dynamics simulations based on tight‐binding quantum chemical calculations as implemented in CREST.[^31^, ^32^] The structures located with CREST have then been subjected to PBE0‐D3(BJ)/def2‐SVP[^23^, ^24^, ^26^, ^33^, ^34^] geometry optimization. The nature of all stationary points (minima and transition states) was verified through the computation of the vibrational frequencies. The thermal corrections to the Gibbs free energies were combined with the single point energies calculated at the PBE0‐D3(BJ)/def2‐TZVP level of theory to yield Gibbs free energies (“*G_298_”*) at 298.15 K. All energies are reported in kcal/mol. The energy profiles were constructed using the most stable conformation (the global minimum) of each intermediate and transition state. The DFT calculations have been performed with the Gaussian 16^[35]^ program package. The polarizable continuum model (PCM) with SMD parameters for dichloromethane[^27^, ^28^, ^36^] was applied to consider solvent effects for both geometries and energies. Free energies in solution have been corrected to a reference state of 1 mol/l at 298.15 K through the addition of RTln(24.46)=+7.925 kJ/mol to the gas phase (1 atm) free energies.

**Quench studies using** ^**18**^ **O‐labeld water**: Alcohol **1 a** (0.2 mmol, 25.6 mg, 1.0 eq.) was weighted into a vial, diluted with 2 mL 1,2‐dichloroethane and brought to the reaction temperature using an oil bath. Aldehyde (0.24 mmol, 33.9 mg, 1.2 eq.) and FeCl_3_ (0.01 mmol, 5 mol %) were dissolved in a minimum amount of 1,2‐dichloroethane, then added to the reaction mixture. The reaction was heated for 10 minutes at 100 °C. ^18^O‐Water (0.2 mL) was added and the reaction was allowed to cool to room temperature with stirring. The reaction mixture was filtered over a short pad of silica, eluted with dichloromethane and the solvent was removed under reduced pressure to obtain the crude product. Purification by flash column chromatography afforded the pure product (0.09 mmol, 24.9 mg, 47 %, 5 : 1 ratio ^18^O/O (**8’**/**8**)).

The spectroscopic data of **8** matched the reported data.^[16]^

^**1**^ **H‐NMR (400 MHz, CDCl_3_)**: δ 7.37–7.29 (m, 2H), 7.25 (d, *J*=7.6 Hz, 3H), 3.92–3.60 (m, 1H), 2.84 (dt, *J*=15.4, 8.0 Hz, 1H), 2.72 (dt, *J*=13.9, 7.9 Hz, 1H), 2.45 (t, *J*=7.3 Hz, 2H), 2.18 (s, 3H), 1.89–1.77 (m, 2H), 1.60 (dddt, *J*=43.8, 17.9, 9.6, 4.6 Hz, 4H), 1.43 (s, 1H), 1.37–1.12 (m, 3H), 0.94 (d, *J*=6.6 Hz, 3H) ppm.

^**13**^ **C‐NMR (100 MHz, CDCl_3_)**: δ 209.3, 142.28, 128.5 (4 C), 125.9, 69.2, 44.9, 44.0, 40.2, 37.3, 32.3, 30.0, 29.2, 21.2, 19.3 ppm.

**HRMS (ESI^+^)**: exact mass calculated for [M+H]^+^ (C_17_H_27_O^18^O) required *m/z* 265.2048, found *m/z* 265.2056.

**FT‐IR (neat) n_max_**_:_ 2926, 2868.26 1713, 1678, 1495, 1454, 1408, 1360, 1312, 1268, 1250, 1231, 1163, 1116, 1086, 1052, 1031, 991, 920, 864, 746 cm^−1^.

## Conflict of Interests

The authors declare no conflict of interest.

1

## Supporting information

As a service to our authors and readers, this journal provides supporting information supplied by the authors. Such materials are peer reviewed and may be re‐organized for online delivery, but are not copy‐edited or typeset. Technical support issues arising from supporting information (other than missing files) should be addressed to the authors.

Supporting Information

## Data Availability

The data that support the findings of this study are available in the supplementary material of this article.
