# Supplementary material for: Computational Insights into the Mechanism of Lewis Acid‐Catalyzed Alkene‐Aldehyde Coupling
Source: Chempluschem. 2025 Feb 11;90(4):e202400751. doi: 10.1002/cplu.202400751 (PMC11997734; doi:10.1002/cplu.202400751)

ChemPlusChem

Supporting Information

Computational Insights into the Mechanism of Lewis Acid-Catalyzed Alkene-Aldehyde Coupling

Ricardo Meyrelles, Bogdan R. Brutiu, and Boris Maryasin*

Table of contents

Computational analysis 2

Energy profile for the coupling of alkenes and aldehydes 2

Exploration of steric clash in the enantioselectivity of the cyclization step 2

Atomic coordinates 3

Experimental section 7

Quench studies using ^{18}O-labeld water 8

NMR Spectra 9

Computational analysis

Energy profile for the coupling of alkenes and aldehydes

The Gibbs free energy profile corresponding to the catalytic cycle shown in Scheme 2 of the main text is presented in Scheme S-1. The depicted transition state structures correspond to the mechanistic steps highlighted in the main text: the nucleophilic attack on the activated aldehyde (**TS$_{AB}$**), the proton transfer (**TS$_{BC}$**), the cyclization step (**TS$_{CD}$**) and finally the [1,5]-hydride shift (**TS$_{DE}$**).

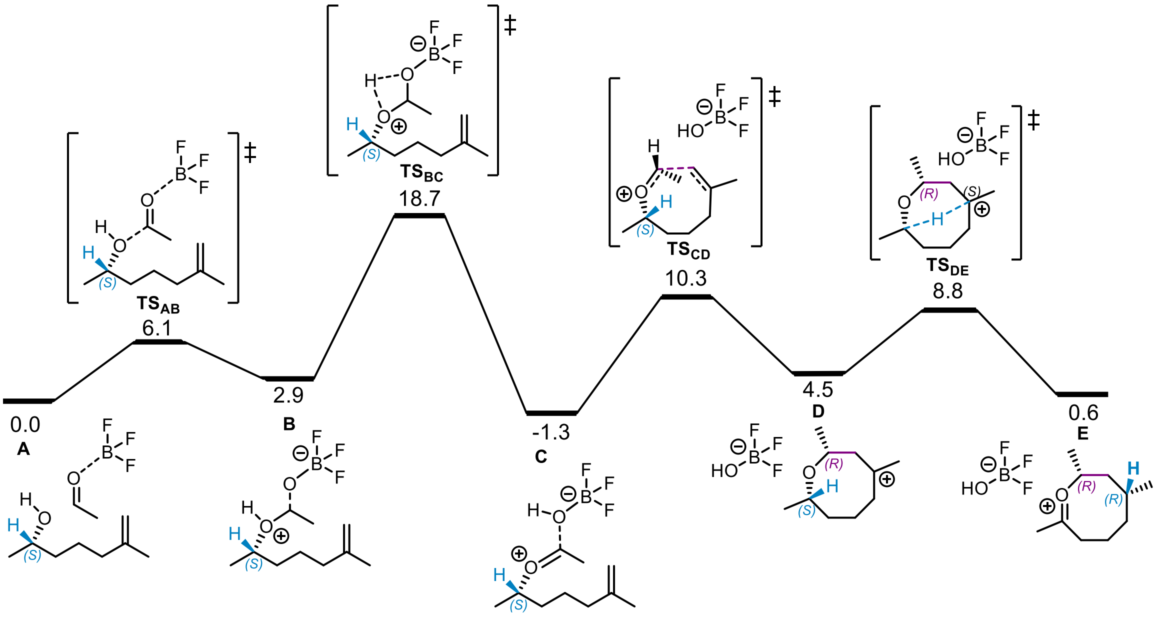

*Scheme S1 – Gibbs free energy profile (in kcal/mol) for the studied Lewis acid catalyzed coupling reaction of an alkene and an aldehyde. The reactant complex **A** serves as the reference (0.0 kcal/mol)*

Exploration of steric clash in the enantioselectivity of the cyclization step

The computed mechanism for the studied reaction of alkenes and aldehydes highlighted that the experimentally observed enantioselectivity arrives from a kinetic preference in the cyclization step, as discussed in the main text. The transition state leading to forming a *(R)*-configured center, **TS$_{CD}$**, was computed to be 4.6 kcal/mol more stable than the competing transition state, which would lead to a *(S)*-configured center, **TS$_{CD'}$**. The disfavoured transition state structure presents a steric clash between the methyl groups bound to the carbons involved in the C-C coupling. To minimize this clash, the alkyl chain adapts a conformation in which a methyl group is in an axial position, as demonstrated in Scheme S2. As an approach to quantify the steric effects leading to the selectivity, these transition state structures were recomputed with the replacement of two methyl groups by a hydrogen atom, one connected to the double bond and one contained in the alkyl chain, which can adopt an axial or equatorial position.

The substitution of the methyl group of the double bond decreases the kinetic preference of **TS$_{CD}$** over **TS$_{CD'}$** by 0.9 kcal/mol, as can be observed for the $\Delta\Delta G^{\ddagger}$(**TS$_{CDa}$**- **TS$_{CD'a}$**), which becomes -3.5 kcal/mol. The distances at which the C-C bond is formed also become shorter for both the transition state structures, likely due to the decreased electron-rich character of the double bond, which acts as a nucleophile. Replacing the methyl group in the C_3 position leads to nearly isoenergetic transition state structures ($\Delta\Delta G^{\ddagger}$(**TS$_{CDb}$**- **TS$_{CD'b}$**) = -0.6 kcal/mol). As a result of the reduction of the steric clash between groups, the C-C bond is formed at very similar distances in both **TS$_{CDb}$** and **TS$_{CD'b}$** (2.08Å and 2.00Å, respectively). As an approximation, it is possible to determine that the steric clash imposed by the methyl groups on the double bond and the alkyl chain contributes roughly 4 kcal/mol in the destabilization of **TS$_{CD'}$**.

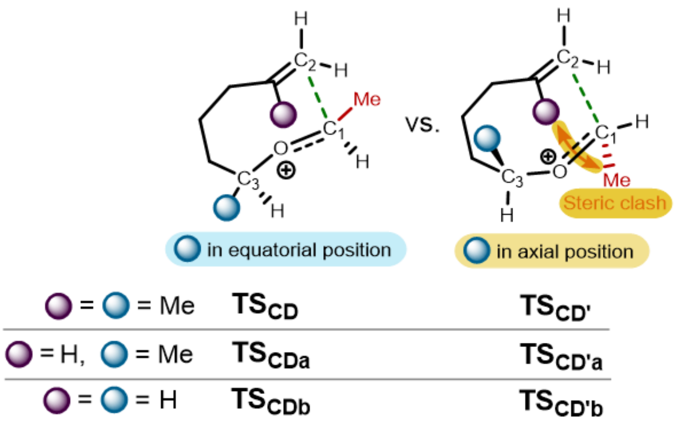

Substituent group	$\Delta\Delta G^{\ddagger}$(**TS**$_{CDx}$ – **TS**$_{CD'x}$) (kcal/mol)	D(C_1–C_2) (Å)	
= Me	-4.6	**TS**$_{CD}$	**TS**$_{CD'}$
= Me		2.17	1.99
= H	-3.5	**TS**$_{CDa}$	**TS**$_{CD'a}$
= Me		2.06	1.94
= H	-0.6	**TS**$_{CDb}$	**TS**$_{CD'b}$
= H		2.08	2.00

*Scheme S2 – Recomputed transition states for the cyclization step with the replacement of the methyl groups by hydrogen atoms and computed parameters: thermodynamic energy difference between the competing transition states ($\Delta\Delta G^{\ddagger}$(**TS**$_{CDx}$ – **TS**$_{CD'x}$) and distance between carbons C_1 and C_2 in the transition state structure.*

Atomic coordinates

Cartesian coordinates of the most stable (ΔG_{298}) conformations, computed at the PBE0-D3(BJ)/def2-TZVP,SMD//PBE0-D3(BJ)/def2-SVP,SMD level of theory:

```
7
2a
C	-0.011023000	1.744249000	-1.828223000
H	-0.855184000	2.005547000	-1.138637000
C	1.054491000	2.781523000	-1.926974000
H	1.850354000	2.468388000	-2.616580000
H	0.606669000	3.731680000	-2.263581000
H	1.469914000	2.972423000	-0.923125000
O	-0.012480000	0.693016000	-2.420396000


25
1a
C	6.632687000	0.719819000	1.621498000
C	5.333968000	0.073623000	1.170626000
C	8.601434000	3.073634000	1.234938000
C	9.871593000	2.652129000	1.161389000
H	7.122398000	0.062283000	2.359648000
H	4.641407000	0.044670000	2.038483000
H	10.483340000	2.831895000	0.270993000
H	7.307152000	0.763060000	0.747473000
H	10.342188000	2.116956000	1.992898000
C	6.448249000	2.112562000	2.204342000
H	5.819540000	2.702533000	1.518863000
H	5.884341000	2.043456000	3.150436000
C	7.763099000	2.847493000	2.467288000
H	8.358044000	2.297468000	3.215157000
H	7.527188000	3.832014000	2.911590000
O	4.791738000	0.896556000	0.154113000
C	5.537476000	-1.345118000	0.669618000
H	4.582866000	-1.782361000	0.333509000
H	5.942866000	-1.994566000	1.461161000
H	6.237009000	-1.354422000	-0.181948000
C	7.938012000	3.790285000	0.096597000
H	7.484654000	4.737262000	0.436146000
H	7.117021000	3.186515000	-0.325982000
H	8.647149000	4.012819000	-0.713699000
H	3.950943000	0.509868000	-0.120998000


32
3a
C	5.007828000	-0.432666000	2.388338000
C	5.982003000	0.672432000	2.071249000
O	6.231876000	0.992464000	0.921813000
C	6.612634000	1.382913000	3.228588000
H	7.212502000	2.235099000	2.884313000
H	7.250036000	0.675924000	3.785191000
H	5.833070000	1.721065000	3.930393000
C	3.547523000	0.027816000	2.252742000
C	3.129139000	0.473893000	0.855160000
C	3.289009000	-0.570831000	-0.256314000
C	2.330855000	-1.741123000	-0.062185000
H	1.282499000	-1.406002000	-0.143686000
H	2.451201000	-2.216532000	0.923953000
H	2.491750000	-2.519662000	-0.824950000
C	3.121650000	0.059049000	-1.645269000
C	4.388507000	0.681213000	-2.235108000
C	4.129867000	1.267500000	-3.609107000
H	3.734924000	0.509615000	-4.302264000
H	5.059813000	1.677286000	-4.031981000
H	3.397054000	2.088214000	-3.540210000
O	4.930707000	1.709985000	-1.438061000
H	5.365186000	1.317841000	-0.661923000
H	5.133488000	-0.135866000	-2.341661000
H	2.783978000	-0.715678000	-2.354698000
H	2.322394000	0.822845000	-1.617276000
H	4.318744000	-0.970522000	-0.208371000
H	3.684589000	1.379470000	0.571674000
H	2.067055000	0.777272000	0.892255000
H	3.366882000	0.854915000	2.960269000
H	2.909072000	-0.802680000	2.595091000
H	5.177468000	-0.794543000	3.413055000
H	5.206258000	-1.266970000	1.697448000


41
A
C	6.610031000	0.678072000	1.649512000
C	5.347807000	-0.025079000	1.184933000
C	8.562357000	3.136773000	1.175529000
C	9.664217000	2.423132000	0.903509000
C	5.987959000	1.758038000	-1.764108000
H	7.025488000	0.108373000	2.497613000
H	4.587522000	0.046278000	1.988195000
H	10.275359000	2.629407000	0.019043000
H	7.355368000	0.613202000	0.839471000
H	9.996313000	1.617094000	1.566028000
H	6.780712000	1.898747000	-1.016619000
C	6.402318000	2.126689000	2.064748000
H	5.863080000	2.666059000	1.268893000
H	5.746486000	2.159502000	2.951486000
C	7.710741000	2.856392000	2.387581000
H	8.285918000	2.260297000	3.115110000
H	7.468870000	3.815283000	2.877933000
O	4.853422000	0.681265000	0.047192000
C	4.932768000	2.765316000	-1.902678000
H	4.105477000	2.404593000	-2.527117000
H	5.403531000	3.634264000	-2.397671000
H	4.587840000	3.099625000	-0.916941000
C	5.581152000	-1.486262000	0.857165000
H	4.654138000	-1.959494000	0.493721000
H	5.906763000	-2.036671000	1.752935000
H	6.350734000	-1.594497000	0.078921000
C	8.101844000	4.254920000	0.288048000
H	8.117340000	5.212627000	0.836629000
H	7.059164000	4.104478000	-0.037668000
H	8.729057000	4.358683000	-0.608021000
B	7.266057000	-0.193298000	-2.571885000
O	6.098971000	0.836242000	-2.592382000
H	4.110877000	0.183910000	-0.324367000
F	8.085601000	0.109446000	-1.499005000
F	6.669469000	-1.420526000	-2.440696000
F	7.913276000	-0.035847000	-3.771709000
C	9.688112000	2.514428000	-3.050783000
H	9.944936000	1.856363000	-3.887420000
H	9.364236000	1.937107000	-2.178753000
Cl	11.126531000	3.450424000	-2.608345000
Cl	8.346540000	3.558651000	-3.565141000


41
TSAB
C	6.650949000	0.780161000	1.512441000
C	5.415013000	0.092801000	0.980257000
C	8.668348000	3.167715000	1.259247000
C	9.795736000	2.465896000	1.075965000
C	5.759463000	1.471481000	-1.495923000
H	7.009530000	0.158562000	2.349376000
H	4.603428000	0.168488000	1.722198000
H	10.462038000	2.665815000	0.230704000
H	7.443552000	0.735554000	0.749477000
H	10.089571000	1.672350000	1.770928000
H	6.648367000	1.808644000	-0.947095000
C	6.433684000	2.205740000	1.997312000
H	5.938394000	2.803084000	1.214083000
H	5.740615000	2.197035000	2.855044000
C	7.739080000	2.890278000	2.413412000
H	8.253037000	2.263853000	3.160756000
H	7.492020000	3.845500000	2.908307000
O	4.886472000	0.879000000	-0.151563000
C	4.826344000	2.551208000	-1.934956000
H	3.894184000	2.124967000	-2.334574000
H	5.328515000	3.094017000	-2.749794000
```

H 4.607814000 3.255022000 -1.122645000
C 5.624666000 -1.347702000 0.584090000
H 4.713094000 -1.774806000 0.137597000
H 5.857786000 -1.931571000 1.486620000
H 6.452653000 -1.458102000 -0.127126000
C 8.251896000 4.260218000 0.320253000
H 8.139742000 5.214888000 0.862460000
H 7.270666000 4.045714000 -0.134677000
H 8.976594000 4.404559000 -0.492406000
B 7.206932000 -0.266380000 -2.468400000
O 5.931623000 0.504722000 -2.343382000
H 4.095670000 0.434055000 -0.511814000
F 8.063857000 0.068240000 -1.411336000
F 6.896257000 -1.616314000 -2.431109000
F 7.794666000 0.068406000 -3.679680000
C 9.559225000 2.507282000 -2.934833000
H 9.764359000 1.835293000 -3.774131000
H 9.235640000 1.944059000 -2.053736000
Cl 11.052307000 3.380301000 -2.536910000
Cl 8.254596000 3.608971000 -3.416549000

41
B
C 7.122245000 -2.730493000 -0.123301000
C 8.283298000 -1.758825000 -0.205907000
C 9.585383000 -2.355186000 0.260217000
H 9.790902000 -3.246118000 -0.350294000
H 10.431508000 -1.666061000 0.133706000
H 9.522348000 -2.667451000 1.313332000
H 8.379555000 -1.358419000 -1.228850000
O 7.942947000 -0.598280000 0.647465000
H 1.700752000 -1.621880000 0.328314000
C 8.925964000 0.672371000 0.683322000
O 8.128728000 1.727527000 0.604311000
B 7.435512000 2.005855000 -0.660436000
F 8.322051000 2.107605000 -1.716826000
F 6.565479000 0.883767000 -0.910374000
F 6.674956000 3.143191000 -0.499506000
C 9.659062000 0.588161000 1.981416000
H 8.946686000 0.683208000 2.812912000
H 10.223743000 -0.346593000 2.076465000
H 10.364725000 1.431200000 2.021064000
H 9.543558000 0.492508000 -0.213333000
C 5.759905000 -2.154969000 -0.483470000
C 4.672348000 -3.224713000 -0.589526000
C 3.298710000 -2.622129000 -0.742071000
C 2.626742000 -2.180961000 0.523547000
H 2.379332000 -3.055995000 1.149541000
H 3.289425000 -1.545950000 1.133661000
C 2.731094000 -2.467691000 -1.945956000
H 1.748567000 -1.997913000 -2.059001000
H 7.128481000 -0.184189000 0.255121000
H 3.230998000 -2.809317000 -2.858586000
H 4.697224000 -3.853773000 0.317609000
H 4.896484000 -3.882883000 -1.444505000
H 5.452707000 -1.420415000 0.279300000
H 5.820000000 -1.609601000 -1.439752000
H 7.370233000 -3.555273000 -0.811836000
H 7.093874000 -3.164894000 0.890593000
C 4.056298000 1.356281000 0.664772000
Cl 2.358277000 1.678502000 1.045175000
Cl 4.938350000 0.681351000 2.054608000
H 4.099799000 0.629255000 -0.152561000
H 4.542912000 2.296062000 0.383392000

41
TSBC
C 6.111136000 2.208188000 -0.460496000
C 7.555384000 1.873565000 -0.160186000
C 3.241009000 2.114019000 0.546791000
C 2.486311000 1.585526000 -0.426213000
C 8.970686000 -0.160119000 -0.573733000
H 6.018718000 2.553452000 -1.504579000
H 7.635745000 1.516304000 0.879067000
H 1.933435000 0.652543000 -0.280531000
H 5.527590000 1.277061000 -0.385259000
H 2.405559000 2.068071000 -1.405865000
H 9.366491000 0.182285000 0.393136000
C 5.526324000 3.235950000 0.497394000
H 5.762982000 2.942478000 1.534549000
H 6.000771000 4.218225000 0.342569000
C 4.012163000 3.391876000 0.337162000
H 3.787781000 3.790822000 -0.665370000
H 3.664015000 4.144562000 1.067056000
O 7.897475000 0.732886000 -1.017511000
C 10.009306000 -0.349341000 -1.624786000
H 10.716072000 -1.120809000 -1.286844000
H 10.559407000 0.588329000 -1.784934000
H 9.544560000 -0.672127000 -2.567100000
C 8.547802000 2.976131000 -0.426297000
H 9.576560000 2.650469000 -0.211234000
H 8.329791000 3.833551000 0.226482000
H 8.489484000 3.308826000 -1.473938000
C 3.382911000 1.455191000 1.886902000
H 3.129447000 2.155661000 2.701034000
H 4.424240000 1.136913000 2.066483000
H 2.737808000 0.569452000 1.975640000
B 7.806627000 -1.768836000 0.969721000
O 8.103034000 -1.271622000 -0.438229000
H 7.341048000 -0.329829000 -0.834313000
F 9.009361000 -1.955483000 1.617728000
F 7.041415000 -0.790579000 1.605679000
F 7.094730000 -2.941572000 0.823863000
C 4.304341000 -1.638684000 -0.076627000
H 4.381591000 -0.757921000 0.568044000
H 4.959084000 -2.445822000 0.266666000
Cl 2.631185000 -2.214662000 -0.043227000
Cl 4.827862000 -1.172791000 -1.711333000

41
C
C 7.001547000 0.999294000 1.193957000
C 5.845558000 0.066488000 0.883049000
C 8.484886000 3.688079000 0.888332000
C 9.706866000 3.283086000 0.514204000
C 5.174923000 1.474831000 -0.978772000
H 7.719157000 0.425276000 1.802160000
H 5.295232000 -0.136814000 1.815529000
H 10.167295000 3.637654000 -0.413337000
H 7.544130000 1.234058000 0.263956000
H 10.286418000 2.580505000 1.121859000
H 5.974663000 2.188648000 -0.743713000
C 6.632354000 2.279197000 1.927764000
H 5.848982000 2.827793000 1.378372000
H 6.187374000 2.023867000 2.904110000
C 7.840789000 3.192473000 2.158237000
H 8.590341000 2.651859000 2.758975000
H 7.515189000 4.062175000 2.755864000
O 4.828348000 0.667024000 0.049909000
C 3.956873000 2.147206000 -1.542544000
H 3.197662000 1.398073000 -1.815095000
H 4.221122000 2.742469000 -2.426179000
H 3.532216000 2.813164000 -0.778448000
C 6.304888000 -1.256706000 0.296084000
H 5.438107000 -1.867080000 0.000963000
H 6.877831000 -1.818069000 1.049255000
H 6.950362000 -1.112201000 -0.582277000
C 7.682898000 4.647258000 0.060685000
H 7.465877000 5.565870000 0.632670000
H 6.705047000 4.219936000 -0.215698000
H 8.206339000 4.927401000 -0.864367000
B 6.849511000 1.239975000 -3.089670000
O 5.827706000 0.652055000 -2.037932000
H 5.210536000 -0.008407000 -2.406214000
F 7.612590000 2.131959000 -2.377833000
F 7.547665000 0.146223000 -3.529928000
F 6.118346000 1.838962000 -4.081145000
C 9.959624000 0.328851000 -1.295705000
H 9.498337000 -0.322435000 -2.044776000
H 9.290887000 1.146180000 -1.010131000
Cl 10.314785000 -0.637432000 0.149765000
Cl 11.417007000 1.048161000 -2.003406000

41
TSCD
C 6.881458000 0.557728000 1.430578000
C 5.440710000 0.905472000 1.095826000
C 7.543227000 3.610024000 0.697617000
C 7.463511000 3.660875000 -0.674975000
C 6.003894000 2.230907000 -0.836178000
H 6.796126000 -0.358166000 2.036605000
H 4.924900000 1.219007000 2.015436000
H 6.948456000 4.506456000 -1.136190000
H 7.423745000 0.249317000 0.522546000
H 8.206761000 3.138799000 -1.283880000
H 6.724352000 1.438157000 -1.056756000
C 7.689814000 1.578552000 2.228273000
H 7.053287000 2.065999000 2.985261000
H 8.467349000 1.037354000 2.788269000
C 8.421094000 2.627294000 1.381846000
H 9.075588000 2.128723000 0.654955000
H 9.071467000 3.215725000 2.054683000
O 5.256097000 2.072358000 0.230186000
C 5.282049000 2.828243000 -1.992706000
H 4.581190000 2.049490000 -2.337456000
H 5.964197000 3.065294000 -2.816498000
H 4.705154000 3.714459000 -1.694448000
C 4.700304000 -0.244618000 0.444106000
H 3.654778000 0.035639000 0.248033000
H 4.703341000 -1.119694000 1.110125000
H 5.175762000 -0.519628000 -0.510774000
C 6.619063000 4.411741000 1.522723000
H 7.027154000 4.628263000 2.519926000
H 5.710849000 3.790137000 1.669332000
H 6.300104000 5.334734000 1.020402000
B 7.440031000 -0.233521000 -2.789820000
O 6.015932000 -0.263717000 -2.756997000
H 5.657917000 -0.018888000 -3.616895000
F 7.946325000 1.084673000 -3.014020000
F 7.916395000 -0.640741000 -1.516195000
F 7.999252000 -1.067266000 -3.773036000
C 10.734028000 0.780268000 -1.418801000
H 11.376184000 0.256603000 -2.135808000
H 9.674025000 0.623098000 -1.641194000
Cl 11.069646000 0.129134000 0.197627000
Cl 11.086873000 2.516737000 -1.533138000

41
D
C 0.964066000 1.929687000 0.078639000
C 1.336949000 0.775123000 0.992774000
C -1.370173000 -0.335215000 0.265343000
C -0.510603000 -1.382934000 -0.288661000
C 0.898496000 -1.495492000 0.415297000
H 0.622458000 2.768129000 0.707357000
H 0.466315000 0.517351000 1.627520000
H -0.994965000 -2.359201000 -0.152541000
H 1.865708000 2.275705000 -0.454190000
H -0.309540000 -1.197000000 -1.353648000
H 0.714407000 -1.647641000 1.491614000
C -0.106123000 1.563654000 -0.941801000
H -0.377198000 2.454216000 -1.527681000
H 0.303182000 0.833222000 -1.656159000
C -1.374764000 1.021090000 -0.300257000
H -2.160679000 0.893530000 -1.082338000
H -1.819863000 1.726482000 0.419380000
O 1.669706000 -0.357717000 0.201210000
C 1.638968000 -2.685253000 -0.150497000
H 2.611797000 -2.774178000 0.354760000
H 1.815848000 -2.558725000 -1.229586000
H 1.073418000 -3.613350000 0.012004000
C 2.489187000 1.095270000 1.923639000
H 2.262881000 1.985318000 2.530063000
H 3.403300000 1.293858000 1.342499000
H 2.671351000 0.251334000 2.603075000
C -2.238417000 -0.631400000 1.369982000
H -1.552524000 -0.466625000 2.271231000
H -2.544338000 -1.681779000 1.439713000
H -3.057105000 0.088072000 1.501792000
O -0.675566000 -0.270759000 3.833729000
B -0.006784000 -1.526272000 4.123739000
H -0.079457000 0.472690000 3.984401000
F -0.117407000 -1.875233000 5.477006000
F 1.364633000 -1.442370000 3.794356000
F -0.604822000 -2.531373000 3.342403000
C -2.443834000 2.372291000 3.880346000
H -2.784939000 2.852583000 4.804288000
H -2.326628000 1.288860000 4.002179000
Cl -3.648885000 2.689895000 2.618894000
Cl -0.846651000 3.035418000 3.473600000

41
TSDE
C 0.728859000 2.258200000 0.175411000
C 1.071104000 1.007930000 0.944975000
C -1.265075000 -0.020291000 0.427093000
C -0.509696000 -1.050446000 -0.313194000
C 0.939317000 -1.222686000 0.226492000
H 0.417237000 3.032837000 0.893519000
H 0.061990000 0.608382000 1.325434000
H -1.014790000 -2.018145000 -0.166213000
H 1.632299000 2.623808000 -0.339893000
H -0.433800000 -0.806197000 -1.382696000
H 0.877596000 -1.491888000 1.297781000
C -0.364254000 1.982097000 -0.851095000
H -0.714897000 2.923357000 -1.298008000
H 0.040932000 1.374931000 -1.671114000
C -1.560635000 1.283099000 -0.203416000
H -2.285115000 1.019713000 -1.002735000
H -2.083664000 1.938680000 0.507805000
O 1.584408000 0.022146000 0.093260000
C 1.710222000 -2.273536000 -0.527848000
H 2.725764000 -2.349863000 -0.112174000
H 1.783207000 -2.025913000 -1.596704000
H 1.217940000 -3.250364000 -0.420164000
C 1.923644000 1.175375000 2.182635000
H 1.474442000 1.898581000 2.876506000
H 2.913726000 1.552803000 1.882378000
H 2.058003000 0.215028000 2.700648000
C -2.027201000 -0.437903000 1.618228000
H -1.545659000 -1.264406000 2.158920000
H -2.980986000 -0.833664000 1.213653000
H -2.281335000 0.398175000 2.281629000
O 2.214334000 0.896829000 -2.626912000
B 1.577186000 0.102032000 -3.614157000
H 2.097242000 0.511134000 -1.748779000
F 0.173038000 -0.071682000 -3.354068000
F 2.116020000 -1.214483000 -3.693659000
F 1.704970000 0.713192000 -4.871431000
C -1.288033000 2.978972000 3.765742000
H -1.490010000 2.913766000 4.840692000
H -0.851589000 2.046964000 3.388449000
Cl -2.829511000 3.254175000 2.932312000
Cl -0.127786000 4.290340000 3.498117000

41
E
C 0.788344000 2.217032000 -0.040388000
C 1.223215000 0.959201000 0.589473000
C -1.479618000 -0.016662000 0.439626000
C -0.785664000 -1.183805000 -0.289990000
C 0.680231000 -1.358912000 0.081173000
H 0.571935000 2.965096000 0.734752000
H -0.889370000 0.204847000 1.353418000
H -1.279250000 -2.134649000 -0.033318000
H 1.638419000 2.581000000 -0.648783000
H -0.857319000 -1.067384000 -1.383267000
H 0.792287000 -1.542134000 1.160479000
C -0.382504000 1.929588000 -0.994609000
H -0.676584000 2.887787000 -1.449236000
H 0.005900000 1.305658000 -1.810267000
C -1.617407000 1.294690000 -0.357063000
H -2.347506000 1.121064000 -1.167065000
H -2.090182000 2.027229000 0.319071000
O 1.233768000 -0.026191000 -0.210886000
C 1.443297000 -2.360207000 -0.736318000
H 2.481404000 -2.448390000 -0.384625000
H 1.447253000 -2.063107000 -1.793806000
H 0.960934000 -3.342919000 -0.634241000
C 1.642485000 0.860981000 1.990447000
H 0.948926000 1.409508000 2.643852000
H 2.594563000 1.425896000 2.031564000
H 1.815493000 -0.162429000 2.339107000
C -2.861300000 -0.444014000 0.930783000
H -2.794567000 -1.300783000 1.618947000
H -3.496461000 -0.743105000 0.080751000
H -3.373689000 0.377524000 1.453852000
O 3.309336000 -0.303836000 -2.682302000
B 2.338840000 0.495391000 -3.350496000
H 3.130969000 -0.292622000 -1.738055000
F 2.212758000 1.787515000 -2.759004000
F 1.035841000 -0.091448000 -3.327187000
F 2.709774000 0.657652000 -4.695388000
C -2.228137000 2.407168000 3.541042000
H -2.492814000 2.312506000 4.600112000
H -1.967233000 1.435044000 3.108471000
Cl -3.629847000 3.051231000 2.672371000
Cl -0.810942000 3.469380000 3.422547000

41
F
C 8.155268000 0.818589000 0.675411000
C 7.554700000 1.119194000 -0.688137000
C 7.191821000 3.173738000 2.395637000

C	6.742022000	3.883087000	1.108869000
C	6.154369000	3.013404000	-0.004410000
H	7.308458000	0.689467000	1.366777000
H	6.527777000	2.299598000	2.527022000
H	5.945659000	4.592887000	1.385173000
H	8.640448000	-0.165519000	0.618338000
H	7.558113000	4.493726000	0.683847000
H	5.564266000	2.199813000	0.452704000
C	9.108971000	1.913272000	1.157489000
H	10.100599000	1.483905000	1.360052000
H	9.264922000	2.634669000	0.342363000
C	8.640916000	2.674186000	2.391777000
H	9.310870000	3.541291000	2.531586000
H	8.777623000	2.041869000	3.286731000
O	7.166342000	2.444118000	-0.840389000
C	5.256901000	3.810300000	-0.930851000
H	4.853255000	3.164489000	-1.724453000
H	5.826681000	4.625176000	-1.405469000
H	4.415780000	4.251841000	-0.376816000
C	6.498287000	0.133788000	-1.126770000
H	6.162037000	0.380275000	-2.143400000
H	5.640649000	0.180450000	-0.441266000
H	6.899208000	-0.887674000	-1.112850000
C	6.981144000	4.079661000	3.605365000
H	7.562075000	5.012043000	3.503651000
H	7.305976000	3.587526000	4.536220000
H	5.921525000	4.356977000	3.722991000
B	9.809051000	0.070378000	-1.874429000
O	8.619348000	1.089001000	-1.713265000
H	8.839520000	2.016952000	-1.951101000
F	10.822261000	0.483082000	-1.035183000
F	9.305508000	-1.154885000	-1.531483000
F	10.160437000	0.177336000	-3.192185000
C	11.333930000	3.768842000	-1.461646000
H	12.188367000	4.105428000	-2.059036000
H	11.437420000	2.719353000	-1.163613000
Cl	11.216692000	4.777835000	-0.018353000
Cl	9.882738000	3.900060000	-2.494953000

41

TSFG

C	8.467952000	0.644360000	0.846402000
C	7.694368000	0.935724000	-0.429304000
C	7.103145000	3.011226000	2.308352000
C	6.843103000	3.933663000	1.106288000
C	6.425525000	3.243254000	-0.176466000
H	7.749325000	0.294225000	1.602727000
H	6.503404000	2.093324000	2.162592000
H	6.018244000	4.621722000	1.352772000
H	9.108409000	-0.219607000	0.624314000
H	7.723207000	4.565730000	0.899546000
H	5.564347000	2.583116000	0.012073000
C	9.273980000	1.823947000	1.387582000
H	10.228134000	1.450688000	1.788937000
H	9.546166000	2.496731000	0.562923000
C	8.568954000	2.598996000	2.500964000
H	9.157201000	3.507302000	2.723602000
H	8.607265000	1.984827000	3.417881000
O	7.505558000	2.387066000	-0.627757000
C	6.112210000	4.192935000	-1.307797000
H	5.830769000	3.641850000	-2.217108000
H	6.979091000	4.831105000	-1.533534000
H	5.270281000	4.838340000	-1.019869000
C	6.416007000	0.164045000	-0.583007000
H	5.915237000	0.429238000	-1.524522000
H	5.739507000	0.332805000	0.265967000
H	6.675087000	-0.904557000	-0.609679000
C	6.603498000	3.670510000	3.591474000
H	7.134477000	4.619640000	3.776490000
H	6.767286000	3.022158000	4.466732000
H	5.526910000	3.896006000	3.534252000
B	9.738448000	0.138178000	-1.878198000
O	8.450983000	0.889077000	-1.632970000
H	8.088528000	2.120332000	-1.613784000
F	10.778267000	0.755837000	-1.181880000
F	9.565058000	-1.164233000	-1.434651000
F	9.955981000	0.196967000	-3.236742000
C	10.710729000	3.919802000	-1.904004000
H	11.620905000	4.264892000	-2.406939000
H	10.839554000	2.914443000	-1.485989000
Cl	10.346835000	5.050627000	-0.588686000
Cl	9.414880000	3.847915000	-3.117407000

41

G

C	8.716474000	0.023972000	1.174167000
C	8.337297000	0.393598000	-0.217475000
C	7.027428000	2.622539000	1.981193000
C	7.681968000	3.783893000	1.230811000
C	6.886176000	4.284832000	0.037005000
H	7.890207000	-0.546036000	1.620922000
H	6.637198000	1.926872000	1.218862000
H	7.856184000	4.623516000	1.925648000
H	9.563668000	-0.671091000	1.038479000
H	8.669648000	3.481543000	0.848255000
H	5.878666000	4.597919000	0.375272000
C	9.164961000	1.153305000	2.118950000
H	9.826437000	0.694335000	2.868562000
H	9.790961000	1.872614000	1.574259000
C	8.029063000	1.859899000	2.848953000
H	8.474877000	2.561308000	3.577331000
H	7.477888000	1.113175000	3.447459000
O	6.757880000	3.186013000	-0.852593000
C	7.555584000	5.464216000	-0.644509000
H	6.997925000	5.765507000	-1.545988000
H	8.582273000	5.206931000	-0.949804000
H	7.601656000	6.333812000	0.029467000
C	7.037964000	-0.041450000	-0.765951000
H	6.849812000	0.399192000	-1.750856000
H	6.223880000	0.175026000	-0.061025000
H	7.091079000	-1.142595000	-0.851284000
C	5.844932000	3.087619000	2.823305000
H	6.178503000	3.768797000	3.624682000
H	5.334737000	2.235837000	3.300992000
H	5.096203000	3.624942000	2.220789000
B	10.604943000	1.431224000	-0.909220000
O	9.111607000	0.975989000	-1.004302000
H	6.196322000	3.450466000	-1.593164000
F	10.580906000	2.742301000	-0.493304000
F	11.269972000	0.603332000	-0.038724000
F	11.058301000	1.299445000	-2.198783000
C	8.600666000	3.000004000	-3.406187000
H	9.335818000	2.232684000	-3.665604000
H	8.437728000	3.069888000	-2.325947000
Cl	9.186887000	4.562426000	-3.999819000
Cl	7.037481000	2.546394000	-4.127078000

30

TSCD'

C	0.289173000	2.020655000	-0.445170000
C	0.967434000	1.067245000	0.524822000
C	-1.710891000	-0.627707000	0.096704000
C	-1.010586000	-1.572604000	-0.612948000
C	0.993641000	-1.285696000	0.168209000
H	-0.083957000	2.862575000	0.161723000
H	0.255351000	0.717518000	1.288260000
H	-1.067901000	-2.614711000	-0.291095000
H	1.047273000	2.435756000	-1.129849000
H	-0.723765000	-1.388857000	-1.650567000
H	0.664832000	-1.378036000	1.211156000
C	-0.858993000	1.420211000	-1.264145000
H	-1.314128000	2.231813000	-1.851713000
H	-0.459247000	0.706660000	-2.001447000
C	-1.960037000	0.762647000	-0.423125000
H	-2.874703000	0.667095000	-1.040143000
H	-2.241268000	1.421200000	0.414503000
O	1.387312000	-0.118219000	-0.233979000
C	1.659172000	-2.448058000	-0.475333000
H	2.680950000	-2.487479000	-0.053609000
H	1.741543000	-2.316210000	-1.562864000
H	1.151481000	-3.389173000	-0.233899000
C	2.199111000	1.638191000	1.199253000
H	1.918256000	2.532549000	1.775453000
H	2.953399000	1.927612000	0.451486000
H	2.642542000	0.905681000	1.890470000
C	-2.290846000	-0.950603000	1.431297000
H	-2.142594000	-2.000573000	1.717029000
H	-3.371767000	-0.723989000	1.426621000
H	-1.849299000	-0.293271000	2.200996000

30

TSCD''

C	6.698869000	0.428840000	1.852440000
C	5.610686000	0.355295000	0.780185000
C	7.505420000	3.468962000	0.335644000
C	7.298298000	2.860668000	-0.891041000
C	5.391451000	2.335344000	-0.656936000
H	6.253515000	0.877286000	2.755723000
H	4.719608000	-0.084790000	1.251540000
H	7.193766000	3.491444000	-1.777167000
H	6.941404000	-0.614107000	2.113785000
H	7.711905000	1.866481000	-1.059891000
H	5.493191000	1.698745000	-1.543689000
C	7.998857000	1.174124000	1.520267000
H	8.730983000	0.890007000	2.291325000
H	8.427269000	0.820759000	0.569222000
C	7.910823000	2.704659000	1.548308000
H	8.910552000	3.121607000	1.786678000
H	7.282780000	3.043292000	2.392749000
O	5.165857000	1.721036000	0.487992000
C	4.560886000	3.565718000	-0.837955000
H	3.524935000	3.228640000	-1.017231000
H	4.888974000	4.148933000	-1.707339000
H	4.558864000	4.193490000	0.063392000
C	5.954432000	-0.470794000	-0.445693000
H	5.120876000	-0.490811000	-1.164584000
H	6.127072000	-1.505237000	-0.113561000
H	6.863887000	-0.132738000	-0.962075000
C	7.252385000	4.920736000	0.525124000
H	8.112103000	5.383543000	1.038636000
H	6.390752000	5.054393000	1.204212000
H	7.059450000	5.446311000	-0.418029000

30

TSDE'

C	0.707841000	2.249102000	0.219775000
C	1.074361000	1.007547000	0.999370000
C	-1.333599000	-0.060050000	0.391047000
C	-0.544632000	-1.077871000	-0.309569000
C	0.922166000	-1.207952000	0.239133000
H	0.335102000	3.008766000	0.925599000
H	0.115176000	0.611779000	1.448615000
H	-1.013846000	-2.060591000	-0.146787000
H	1.611991000	2.662655000	-0.256646000
H	-0.470678000	-0.855815000	-1.382938000
H	0.847650000	-1.505627000	1.302633000
C	-0.338822000	1.937145000	-0.842250000
H	-0.672521000	2.857806000	-1.341297000
H	0.102373000	1.296125000	-1.620098000
C	-1.564051000	1.258583000	-0.224372000
H	-2.284000000	1.021158000	-1.037951000
H	-2.086530000	1.924026000	0.478010000
O	1.561600000	0.024695000	0.119777000
C	1.692062000	-2.243116000	-0.540280000
H	2.707706000	-2.328830000	-0.127422000
H	1.767393000	-1.954331000	-1.599304000
H	1.202074000	-3.224226000	-0.472258000
C	2.015698000	1.217251000	2.167162000
H	1.594827000	1.936916000	2.883786000
H	2.976595000	1.608719000	1.798931000
H	2.202476000	0.268439000	2.690893000
C	-2.102606000	-0.453861000	1.576325000
H	-1.663654000	-1.313690000	2.100769000
H	-3.078628000	-0.794809000	1.169814000
H	-2.313437000	0.386333000	2.250374000

30

TSDE''

C	-0.012314000	2.386829000	0.041205000
C	0.581787000	1.143528000	0.654397000
C	-1.524657000	0.061942000	0.372126000
C	-0.827591000	-1.167401000	0.901132000
C	0.713956000	-1.175357000	0.454770000
H	0.580367000	3.277116000	0.299413000
H	-0.553741000	0.679173000	0.461900000
H	-0.875564000	-1.204550000	1.997385000
H	-0.036856000	2.277811000	-1.051569000
H	-1.279935000	-2.085457000	0.500517000
H	1.307405000	-1.397541000	1.353437000
C	-1.473458000	2.552643000	0.632180000
H	-1.453887000	3.258590000	1.474208000
H	-2.079672000	3.028059000	-0.151558000
C	-2.163974000	1.221038000	1.126083000
H	-3.244849000	1.283944000	0.930777000
H	-2.018461000	1.093878000	2.207974000
O	1.132129000	0.142244000	-0.039591000
C	1.053509000	-2.170572000	-0.621989000
H	2.122160000	-2.109294000	-0.873376000
H	0.462956000	-2.005778000	-1.534617000
H	0.849100000	-3.185649000	-0.252410000
C	0.877773000	1.219709000	2.114260000
H	0.243130000	1.952074000	2.625859000
H	1.919507000	1.585995000	2.165423000
H	0.839560000	0.255994000	2.634448000
C	-1.862452000	0.012277000	-1.088881000
H	-1.036432000	-0.382344000	-1.697548000
H	-2.174804000	0.987518000	-1.484795000
H	-2.709646000	-0.685994000	-1.202202000

28

1b

C	5.246118000	-2.335287000	-0.301945000
C	6.578272000	-1.758192000	-0.754071000
C	1.567341000	0.232435000	0.202714000
C	1.515365000	1.295708000	-0.611662000
H	5.119252000	-3.326085000	-0.774743000
H	6.694264000	-0.759666000	-0.281128000
H	1.548896000	2.318196000	-0.220823000
H	5.320669000	-2.519218000	0.783832000
H	1.432965000	1.176731000	-1.697131000
C	2.744599000	-2.022827000	-0.017022000
H	2.581811000	-3.038005000	-0.418217000
H	2.847408000	-2.146469000	1.075286000
C	1.509958000	-1.175231000	-0.331863000
H	0.623921000	-1.677147000	0.097805000
H	1.358082000	-1.141926000	-1.423319000
O	7.577139000	-2.637224000	-0.273458000
C	6.687316000	-1.588081000	-2.259486000
H	7.684315000	-1.205352000	-2.534023000
H	5.944198000	-0.874034000	-2.645686000
H	6.539811000	-2.555494000	-2.767129000
C	1.689963000	0.379338000	1.689970000
H	1.644885000	1.432689000	2.001943000
H	2.641712000	-0.042734000	2.054558000
H	0.887399000	-0.173763000	2.207290000
C	4.037599000	-1.457990000	-0.586659000
H	4.212921000	-0.448692000	-0.172593000
H	3.917026000	-1.318110000	-1.675396000
H	8.431861000	-2.295293000	-0.564765000

35

3b

C	5.723602000	2.635663000	-0.609222000
C	5.149651000	1.920141000	0.589734000
O	5.545641000	0.824547000	0.947226000
C	4.041550000	2.614942000	1.320238000
H	3.313937000	3.044491000	0.614712000
H	3.545731000	1.927285000	2.017455000
H	4.472904000	3.458334000	1.885641000
C	7.116664000	2.178213000	-1.004246000
C	8.194188000	2.677816000	-0.046958000
C	9.576038000	2.066176000	-0.265870000
C	9.630941000	0.534842000	-0.230981000
C	11.043471000	0.036451000	-0.511196000
H	11.752750000	0.409497000	0.247503000
H	11.402823000	0.373083000	-1.497392000
H	11.093154000	-1.064382000	-0.499024000
C	9.078835000	-0.055088000	1.072464000
C	8.348447000	-1.382557000	0.880692000
C	7.909618000	-1.983935000	2.206527000
H	8.770047000	-2.192344000	2.861863000
H	7.235640000	-1.291760000	2.739134000
H	7.365181000	-2.925937000	2.038748000
O	7.251063000	-1.240181000	0.003483000
H	6.676460000	-0.534131000	0.353532000
H	9.031590000	-2.095015000	0.382567000
H	9.894856000	-0.184403000	1.804807000
H	8.359422000	0.637467000	1.540364000
H	8.982374000	0.163785000	-1.042432000
H	10.268648000	2.470137000	0.495307000
H	9.971341000	2.402924000	-1.241426000
H	8.270057000	3.775981000	-0.129881000
H	7.867062000	2.487004000	0.989566000
H	7.120006000	1.078446000	-1.031467000
H	7.341389000	2.517751000	-2.028963000
H	4.997721000	2.444704000	-1.422518000
H	5.676987000	3.723368000	-0.431944000

44

A''

C	6.599288000	2.974961000	-0.402159000
C	7.911409000	2.233107000	-0.233876000
C	2.649037000	1.199945000	0.663708000

C 2.820707000 0.181267000 1.518453000
C 8.779236000 -0.284333000 -0.475832000
H 6.793759000 4.016369000 -0.096911000
H 8.211375000 2.206325000 0.826934000
H 2.575741000 -0.847804000 1.236630000
H 6.348240000 3.002295000 -1.476456000
H 3.211275000 0.345770000 2.528070000
H 9.498741000 0.210606000 0.198228000
C 4.152251000 3.218532000 0.254237000
H 4.321765000 4.252321000 0.600168000
H 3.882624000 3.294980000 -0.813386000
C 2.988401000 2.617678000 1.048714000
H 2.096562000 3.252116000 0.899416000
H 3.230092000 2.651976000 2.123588000
O 7.648196000 0.827439000 -0.614114000
C 9.286256000 -0.539315000 -1.857638000
H 8.473007000 -0.938583000 -2.479431000
H 10.084490000 -1.292895000 -1.787658000
H 9.698764000 0.366417000 -2.317376000
C 9.020536000 2.781860000 -1.091204000
H 9.974318000 2.263071000 -0.923454000
H 9.167162000 3.838378000 -0.824646000
H 8.758450000 2.729198000 -2.158447000
C 2.125560000 0.988981000 -0.725242000
H 1.915949000 -0.070772000 -0.926795000
H 2.848395000 1.337793000 -1.480754000
H 1.200753000 1.569599000 -0.885750000
C 5.432776000 2.411467000 0.394778000
H 5.212027000 1.382425000 0.066131000
H 5.709240000 2.338131000 1.461109000
O 8.165274000 -1.338008000 0.052312000
B 7.680553000 -1.259915000 1.437107000
H 6.953627000 0.481348000 0.009671000
F 8.657906000 -0.785391000 2.293379000
F 6.592787000 -0.306993000 1.427914000
F 7.203094000 -2.493112000 1.806172000
C 4.907401000 -1.976359000 -0.710492000
H 4.867125000 -1.363560000 0.195098000
H 5.745031000 -2.680472000 -0.680587000
Cl 3.400465000 -2.901189000 -0.828520000
Cl 5.147191000 -0.896225000 -2.100195000

44
TSAB''
C 7.718296000 0.750054000 -2.445086000
C 6.853896000 -0.175781000 -1.607397000
C 8.970867000 3.224549000 1.164519000
C 8.088761000 3.155231000 2.171156000
C 4.944102000 -0.590343000 -4.038705000
H 7.726945000 0.377835000 -3.483638000
H 6.817622000 0.208142000 -0.572057000
H 8.333262000 3.521673000 3.173686000
H 7.225816000 1.736074000 -2.470261000
H 7.090339000 2.729904000 2.023210000
H 5.878693000 -1.152449000 -4.171479000
C 9.329990000 1.363973000 -0.518350000
H 8.956835000 0.599172000 0.184842000
H 10.409896000 1.454544000 -0.321698000
C 8.643616000 2.695912000 -0.207766000
H 7.552087000 2.582512000 -0.301983000
H 8.958363000 3.436732000 -0.964283000
O 5.510158000 -0.075108000 -2.111772000
C 4.824804000 0.755818000 -4.621891000
H 3.971155000 1.296493000 -4.193055000
H 4.639843000 0.606892000 -5.701070000
H 5.753601000 1.325951000 -4.513030000
C 7.325809000 -1.614865000 -1.579648000
H 6.628448000 -2.244895000 -1.007647000
H 8.310478000 -1.684188000 -1.094866000
H 7.413246000 -2.029994000 -2.595575000
C 10.337544000 3.816591000 1.344245000
H 10.508882000 4.148816000 2.378300000
H 10.472232000 4.682490000 0.673638000
H 11.126997000 3.093561000 1.079991000
C 9.157752000 0.894873000 -1.960346000
H 9.687842000 -0.064246000 -2.086032000
H 9.671242000 1.607769000 -2.627062000
O 3.889203000 -1.232458000 -3.772474000
B 3.952001000 -2.633522000 -3.163118000
H 4.958834000 -0.731457000 -1.647309000
F 5.052908000 -3.292179000 -3.665581000
F 4.079849000 -2.424828000 -1.784069000
F 2.770748000 -3.248186000 -3.477126000
C 12.839883000 3.953351000 -1.502815000
H 12.718985000 4.436058000 -0.527064000
H 13.723804000 4.333842000 -2.026943000
Cl 13.062344000 2.214875000 -1.240994000
Cl 11.414885000 4.327553000 -2.488420000

44
B''
C 7.721283000 -2.413291000 -1.345474000
C 7.857045000 -0.934089000 -1.635202000
C 8.599406000 -0.640093000 -2.912495000
H 8.018943000 -1.037178000 -3.757434000
H 9.589905000 -1.119256000 -2.909657000
H 8.725070000 0.439836000 -3.075806000
H 6.874637000 -0.439164000 -1.630352000
O 8.626672000 -0.308146000 -0.531786000
H 8.400033000 -0.696458000 0.361792000
C 8.405163000 1.215112000 -0.294757000
O 7.306973000 1.350938000 0.454593000
B 7.280303000 0.894631000 1.846535000
F 7.962315000 1.736216000 2.702729000
F 5.954147000 0.765401000 2.218917000
F 7.912609000 -0.406596000 1.885606000
C 9.715825000 1.702735000 0.255147000
H 9.981799000 1.192752000 1.190788000
H 9.623287000 2.779129000 0.457827000
H 10.512309000 1.547753000 -0.484911000
H 8.216840000 1.586077000 -1.311126000
C 6.890793000 -2.805728000 -0.125468000
C 5.502274000 -2.179322000 -0.094007000
C 4.538112000 -2.879244000 0.864732000
C 3.212732000 -2.164125000 0.959812000
C 2.202224000 -2.454954000 0.128111000
H 2.289344000 -3.252994000 -0.617222000
H 1.254153000 -1.908467000 0.167196000
C 3.110606000 -1.089940000 1.999445000
H 3.972142000 -0.406089000 1.953519000
H 2.187231000 -0.501143000 1.899232000
H 3.128928000 -1.535871000 3.009488000
H 5.000649000 -2.936742000 1.865300000
H 4.385115000 -3.915640000 0.521308000
H 5.062957000 -2.196469000 -1.107029000
H 5.584159000 -1.118188000 0.188579000
H 7.422443000 -2.567018000 0.811639000
H 6.800320000 -3.904711000 -0.135232000
H 8.729880000 -2.856232000 -1.287450000
H 7.249990000 -2.837708000 -2.247115000
C 4.168938000 1.169840000 -0.604229000
Cl 4.886868000 1.458144000 -2.205381000
Cl 3.029236000 2.441857000 -0.131813000
H 4.982854000 1.147280000 0.127268000
H 3.622911000 0.221479000 -0.646869000

44
TSBC''
C 8.316372000 2.173116000 -1.448417000
C 8.935486000 0.953522000 -0.790550000
C 3.658044000 2.473287000 0.666451000
C 2.565346000 2.504139000 -0.110457000
C 8.179767000 -1.365830000 -0.151317000
H 9.149699000 2.849903000 -1.693826000
H 9.063934000 1.114564000 0.293497000
H 1.650289000 1.971619000 0.168291000
H 7.888867000 1.853848000 -2.414955000
H 2.553703000 3.061012000 -1.053361000
H 9.010606000 -1.221098000 0.553893000
C 6.008486000 2.220922000 -0.267822000
H 5.614267000 1.686164000 -1.149641000
H 6.218750000 1.462080000 0.504557000
C 4.928817000 3.170725000 0.257290000
H 4.705870000 3.915014000 -0.524378000
H 5.336127000 3.725012000 1.121976000
O 7.979274000 -0.150116000 -0.939880000
C 8.304315000 -2.579622000 -1.006014000
H 9.237811000 -2.542767000 -1.584343000
H 7.444833000 -2.650986000 -1.686553000
H 8.326201000 -3.465484000 -0.355145000
C 10.236872000 0.529385000 -1.427069000
H 10.641236000 -0.382715000 -0.964271000
H 10.980707000 1.327828000 -1.292931000
H 10.103136000 0.352709000 -2.505392000
C 3.694469000 1.717573000 1.960222000
H 3.887520000 2.404132000 2.802453000
H 4.509262000 0.976330000 1.970769000
H 2.750363000 1.187502000 2.152520000
C 7.291052000 2.956872000 -0.636430000
H 7.769321000 3.334205000 0.284577000
H 7.023777000 3.851478000 -1.224452000
O 6.918730000 -1.250363000 0.484338000
B 6.891614000 -1.124153000 2.000915000
H 6.982634000 -0.220263000 -0.253419000
F 5.559630000 -1.183678000 2.361384000
F 7.626268000 -2.172625000 2.516521000
F 7.457516000 0.099597000 2.336974000
C 3.835745000 -0.906169000 -0.502438000
H 4.455609000 -1.348303000 0.283620000
H 3.779017000 0.183671000 -0.411173000
Cl 4.572310000 -1.275904000 -2.076678000
Cl 2.189189000 -1.542669000 -0.350860000

44
C''
C 2.912343000 -0.904019000 -0.114873000
C 2.549019000 0.057991000 1.005081000
C 6.766434000 -3.256317000 -0.103728000
C 7.253928000 -3.503665000 1.119994000
C 4.188706000 1.759662000 0.521832000
H 1.990351000 -1.440703000 -0.395833000
H 2.346541000 -0.524608000 1.917304000
H 8.204641000 -3.075498000 1.452840000
H 3.211546000 -0.330109000 -1.007855000
H 6.713986000 -4.140411000 1.828785000
H 3.504172000 2.029650000 -0.295711000
C 4.396617000 -2.793947000 -0.922463000
H 3.505076000 -3.316635000 -1.310030000
H 4.756222000 -2.158546000 -1.750099000
C 5.457346000 -3.838587000 -0.568831000
H 5.648269000 -4.457994000 -1.464006000
H 5.062214000 -4.514059000 0.207861000
O 3.659338000 0.884346000 1.413168000
C 4.733892000 2.967268000 1.231121000
H 5.253097000 3.627042000 0.524477000
H 5.425571000 2.661498000 2.030457000
H 3.898558000 3.516005000 1.687899000
C 1.330434000 0.905498000 0.692495000
H 1.140588000 1.625570000 1.502588000
H 0.445952000 0.259146000 0.592914000
H 1.440907000 1.459433000 -0.252983000
C 7.485593000 -2.372245000 -1.078004000
H 8.484656000 -2.094133000 -0.714823000
H 6.922054000 -1.444283000 -1.265424000
H 7.595591000 -2.871274000 -2.055756000
C 3.995630000 -1.908012000 0.247036000
H 4.882099000 -1.372847000 0.622854000
H 3.647125000 -2.538008000 1.085580000
O 5.294711000 1.081055000 -0.202279000
B 5.711402000 1.433015000 -1.673059000
H 6.051071000 0.881661000 0.390140000
F 7.044032000 1.080886000 -1.722063000
F 4.924563000 0.672289000 -2.496935000
F 5.503674000 2.776840000 -1.847580000
C 9.092114000 0.593322000 0.721063000
H 8.739212000 0.536175000 -0.313705000
H 9.517233000 1.576656000 0.950298000
Cl 7.673287000 0.365088000 1.787643000
Cl 10.324426000 -0.636853000 0.994051000

44
TSCD''
C 8.015225000 0.407016000 -2.040793000
C 7.042267000 0.179756000 -0.893634000
C 8.336281000 2.970229000 0.254842000
C 7.207704000 3.552047000 -0.261234000
C 5.678329000 1.978060000 -0.190099000
H 7.748379000 -0.253277000 -2.881441000
H 7.499202000 0.449154000 0.071671000
H 6.560332000 4.107374000 0.423160000
H 7.870380000 1.433127000 -2.408945000
H 7.128410000 3.764478000 -1.331546000
C 9.962513000 1.101973000 -0.511786000
H 9.656569000 0.672665000 0.455756000
H 11.062074000 1.081534000 -0.499230000
C 9.494286000 2.568206000 -0.606135000
H 9.295349000 2.848970000 -1.648698000
H 10.325970000 3.212055000 -0.266483000
O 5.950691000 1.116291000 -1.104224000
H 5.967076000 1.730805000 0.840668000
C 6.465512000 -1.210976000 -0.808662000
H 5.787105000 -1.312081000 0.051503000
H 7.286191000 -1.933657000 -0.687924000
H 5.918755000 -1.459778000 -1.730672000
C 8.475042000 2.769610000 1.724783000
H 7.654259000 3.235145000 2.286605000
H 9.435736000 3.178567000 2.078413000
H 8.496011000 1.692527000 1.966150000
C 9.478881000 0.218736000 -1.663760000
H 9.682053000 -0.836649000 -1.414846000
H 10.076904000 0.444852000 -2.561574000
O 5.623717000 4.181244000 -3.228675000
B 6.614458000 3.650553000 -4.116943000
H 4.852882000 4.455349000 -3.736864000
F 6.846060000 4.501335000 -5.219325000
F 7.831551000 3.516475000 -3.394943000
F 6.272913000 2.376147000 -4.624497000
C 12.968319000 2.010492000 -2.688092000
H 13.892683000 2.006754000 -3.276724000
H 12.629106000 0.990096000 -2.478224000
Cl 13.306245000 2.813614000 -1.141858000
Cl 11.715689000 2.833650000 -3.629582000
C 4.500050000 2.828124000 -0.460514000
H 4.608248000 3.309660000 -1.446354000
H 3.624521000 2.155390000 -0.487363000
H 4.351978000 3.572224000 0.330169000

44
D''
C 7.489545000 0.263811000 -2.757840000
C 7.150602000 0.488390000 -1.284423000
C 9.133917000 2.245939000 -1.033649000
C 8.951853000 3.036363000 0.266522000
C 7.557039000 2.452404000 0.446307000
H 8.429200000 -0.302629000 -2.841899000
H 7.664191000 -0.234788000 -0.635280000
H 9.657876000 2.757766000 1.055768000
H 6.705064000 -0.399579000 -3.152375000
H 8.957392000 4.123007000 0.116302000
C 8.925293000 2.248901000 -3.586176000
H 8.967267000 2.959007000 -4.427503000
H 9.733295000 1.520288000 -3.769743000
C 9.214717000 3.040073000 -2.314764000
H 8.510954000 3.883893000 -2.231805000
H 10.224273000 3.479730000 -2.361263000
O 7.664848000 1.817594000 -0.917097000
H 7.497643000 1.631506000 1.174770000
C 5.672631000 0.484452000 -0.987229000
H 5.481240000 0.643174000 0.082154000
H 5.278827000 -0.508597000 -1.247788000
H 5.135136000 1.239191000 -1.580996000
C 10.078217000 1.083078000 -0.904869000
H 9.828032000 0.460952000 -0.036555000
H 11.078074000 1.508976000 -0.732481000
H 10.124718000 0.460702000 -1.806666000
C 7.581527000 1.516433000 -3.634620000
H 7.416734000 1.198684000 -4.675926000
H 6.758061000 2.212316000 -3.397662000
O 7.084030000 -0.278528000 2.035153000
B 8.441464000 -0.728623000 2.089294000
H 6.497895000 -1.022418000 1.860036000
F 8.813816000 -1.417531000 0.897811000
F 9.275964000 0.403559000 2.220173000
F 8.691520000 -1.600615000 3.167213000
C 12.722427000 4.178443000 -0.563653000
H 13.744078000 4.532036000 -0.742478000
H 12.407682000 3.472139000 -1.340107000
Cl 12.692882000 3.341953000 0.998165000
Cl 11.637500000 5.580077000 -0.611729000
C 6.406223000 3.405391000 0.524140000
H 6.434304000 4.135623000 -0.297934000
H 5.436012000 2.891387000 0.526986000
H 6.499623000 3.954492000 1.474659000

44
TSDE''
C 8.248347000 0.270231000 -2.187145000
C 7.683166000 0.614022000 -0.812834000
C 8.488440000 3.336615000 -0.203148000
C 7.056637000 3.544754000 -0.360101000
C 6.184773000 2.273481000 -0.052019000
H 8.645852000 -0.756576000 -2.178448000
H 8.395429000 1.311047000 -0.293928000
H 6.756453000 4.270607000 0.415607000
H 7.394370000 0.274932000 -2.883566000

H	6.789637000	3.899559000	-1.369453000
C	9.028493000	2.709505000	-2.673663000
H	9.648959000	3.195789000	-3.440885000
H	7.987558000	2.911341000	-2.964176000
C	9.389756000	3.414789000	-1.360385000
H	9.347843000	4.512730000	-1.551954000
H	10.424186000	3.194210000	-1.059898000
O	6.467234000	1.297828000	-0.997907000
H	6.440455000	1.925485000	0.966278000
C	7.500321000	-0.581736000	0.103729000
H	7.067718000	-0.285703000	1.071192000
H	8.464437000	-1.074874000	0.292944000
H	6.825295000	-1.308972000	-0.374626000
C	9.067576000	3.319672000	1.146295000
H	8.333219000	3.081729000	1.927228000
H	9.411341000	4.360551000	1.320032000
H	9.963180000	2.687183000	1.209868000
C	9.337646000	1.210744000	-2.693452000
H	10.267799000	1.031743000	-2.126191000
H	9.563304000	0.924850000	-3.733084000
O	6.298084000	4.607784000	-3.303748000
B	5.489504000	3.756787000	-4.129979000
H	5.800300000	5.402492000	-3.083138000
F	5.080587000	4.410648000	-5.313514000
F	6.241504000	2.617642000	-4.480136000
F	4.313061000	3.330708000	-3.456453000
C	12.583226000	0.668057000	-0.145975000
H	13.467980000	0.109132000	0.179556000
H	12.383995000	0.509211000	-1.211519000
Cl	11.189383000	0.078444000	0.779137000
Cl	12.892600000	2.393098000	0.114756000
C	4.717467000	2.620189000	-0.135383000
H	4.462152000	2.963227000	-1.148581000
H	4.120641000	1.725211000	0.094345000
H	4.463373000	3.406848000	0.588976000

44
E''

C	7.834786000	0.230098000	-2.774814000
C	7.166143000	0.377691000	-1.474184000
C	8.635148000	2.787041000	0.068946000
C	7.169679000	3.263928000	0.005774000
C	6.150165000	2.145587000	-0.108971000
H	8.045062000	-0.826689000	-2.983017000
H	8.650096000	1.686977000	0.182628000
H	6.906951000	3.819380000	0.920255000
H	7.202612000	0.674951000	-3.556934000
H	7.004130000	3.939386000	-0.848559000
C	9.008743000	2.555238000	-2.531603000
H	9.634200000	3.035556000	-3.301120000
H	7.978329000	2.860477000	-2.772316000
C	9.443496000	3.139995000	-1.186790000
H	9.448083000	4.240908000	-1.280205000
H	10.493296000	2.851887000	-1.001487000
O	6.529444000	1.458742000	-1.351414000
H	6.246984000	1.431746000	0.720460000
C	7.304111000	-0.649372000	-0.433846000
H	6.968913000	-0.354798000	0.564776000
H	8.345446000	-1.002442000	-0.411120000
H	6.699239000	-1.504593000	-0.789007000
C	9.335608000	3.342460000	1.305369000
H	8.802279000	3.060202000	2.227161000
H	9.380749000	4.443982000	1.268413000
H	10.366321000	2.964492000	1.382539000
C	9.163033000	1.033839000	-2.691451000
H	9.785194000	0.614405000	-1.883417000
H	9.688679000	0.802590000	-3.629667000
O	5.993815000	4.283433000	-2.998081000
B	5.356802000	3.512703000	-4.022597000
H	5.472150000	5.073698000	-2.824202000
F	5.044473000	4.303848000	-5.154081000
F	6.230720000	2.477122000	-4.431077000
F	4.146809000	2.919911000	-3.580458000
C	12.280088000	0.168572000	-0.049191000
H	13.046081000	-0.598634000	0.109828000
H	11.946200000	0.183777000	-1.092373000
Cl	10.889371000	-0.226596000	0.979780000
Cl	12.988189000	1.745732000	0.332454000
C	4.727248000	2.600771000	-0.287035000
H	4.651247000	3.231215000	-1.183490000
H	4.047740000	1.741849000	-0.388262000
H	4.425730000	3.177670000	0.599173000

27
TSCDa

C	0.291137000	2.022110000	-0.466109000
C	0.946689000	1.051421000	0.492747000
C	-1.666961000	-0.595582000	0.074027000
C	-0.942331000	-1.560975000	-0.574639000
C	0.965759000	-1.282269000	0.152735000
H	-0.061888000	2.870558000	0.143237000
H	0.216335000	0.703835000	1.245927000
H	-1.038352000	-2.591613000	-0.222948000
H	1.054155000	2.423819000	-1.153050000
H	-0.648361000	-1.417121000	-1.618934000
H	0.693493000	-1.366433000	1.213810000
C	-0.866457000	1.441637000	-1.273779000
H	-1.332577000	2.257510000	-1.845331000
H	-0.482852000	0.731555000	-2.023745000
C	-1.937112000	0.779009000	-0.411336000
H	-2.873022000	0.673940000	-0.999213000
H	-2.211414000	1.424574000	0.438236000
O	1.354698000	-0.115903000	-0.265572000
C	1.649749000	-2.438188000	-0.477461000
H	2.670096000	-2.450715000	-0.054227000
H	1.728971000	-2.319509000	-1.566148000
H	1.161831000	-3.386056000	-0.223609000
C	2.163839000	1.601920000	1.198437000
H	1.878481000	2.485245000	1.787720000
H	2.932325000	1.901486000	0.470168000
H	2.591651000	0.854054000	1.882081000
H	-2.116612000	-0.865762000	1.038136000

27
TSCD'a

C	6.722364000	0.402779000	1.848068000
C	5.638272000	0.357877000	0.777087000
C	7.488215000	3.393505000	0.259632000
C	7.302409000	2.781022000	-0.954596000
C	5.430695000	2.350329000	-0.624069000
H	6.266983000	0.800523000	2.769780000
H	4.742137000	-0.081534000	1.240272000
H	7.198419000	3.396518000	-1.851826000
H	6.995214000	-0.642182000	2.065929000
H	7.687902000	1.770986000	-1.106853000
H	5.472607000	1.721055000	-1.523873000
C	7.988239000	1.202714000	1.546592000
H	8.706657000	0.977647000	2.348624000
H	8.468273000	0.852426000	0.618340000
C	7.799575000	2.716640000	1.520163000
H	8.711801000	3.238823000	1.879327000
H	7.040790000	3.044440000	2.259241000
O	5.220841000	1.724831000	0.513004000
C	4.631750000	3.604618000	-0.754907000
H	3.585437000	3.302579000	-0.927744000
H	4.962006000	4.203951000	-1.612316000
H	4.668820000	4.204965000	0.163665000
C	5.970727000	-0.451201000	-0.455164000
H	5.144004000	-0.441650000	-1.181735000
H	6.112261000	-1.494577000	-0.138595000
H	6.894866000	-0.133593000	-0.958212000
H	7.305748000	4.472892000	0.332842000

24
TSCDb

C	0.280271000	2.016369000	-0.469270000
C	0.934475000	1.038107000	0.474206000
C	-1.672185000	-0.612190000	0.053896000
C	-0.938488000	-1.554510000	-0.615720000
C	0.975055000	-1.291054000	0.157828000
H	-0.049167000	2.865078000	0.153651000
H	0.258202000	0.714363000	1.283691000
H	-1.028179000	-2.595139000	-0.293274000
H	1.033413000	2.415404000	-1.167471000
H	-0.624921000	-1.378878000	-1.649376000
H	0.674132000	-1.379882000	1.210346000
C	-0.898753000	1.455192000	-1.261996000
H	-1.374124000	2.283465000	-1.807538000
H	-0.533330000	0.759675000	-2.034657000
C	-1.948873000	0.775004000	-0.390811000
H	-2.900543000	0.687598000	-0.955834000
H	-2.201593000	1.399107000	0.481578000
O	1.374308000	-0.124178000	-0.247100000
C	1.661747000	-2.445160000	-0.468531000
H	2.675561000	-2.465334000	-0.029478000
H	1.758792000	-2.321013000	-1.555154000
H	1.165576000	-3.391679000	-0.226242000
H	1.829181000	1.474440000	0.942594000
H	-2.129662000	-0.912996000	1.005141000

24
TSCD'b

C	6.727775000	0.394259000	1.845643000
C	5.696341000	0.412790000	0.740329000
C	7.499561000	3.334378000	0.191526000
C	7.296120000	2.659426000	-0.981810000
C	5.357386000	2.364840000	-0.569806000
H	6.238350000	0.721296000	2.778492000
H	4.831563000	-0.210745000	1.009575000
H	7.194636000	3.219334000	-1.915190000
H	7.010434000	-0.660009000	1.998132000
H	7.598263000	1.612792000	-1.074221000
H	5.365969000	1.721809000	-1.462699000
C	7.988642000	1.224581000	1.614145000
H	8.654778000	1.050376000	2.472174000
H	8.537762000	0.851460000	0.733511000
C	7.773966000	2.732081000	1.502748000
H	8.656556000	3.286143000	1.881357000
H	6.968642000	3.078065000	2.183243000
O	5.194002000	1.749239000	0.574884000
C	4.625125000	3.653671000	-0.684188000
H	3.561430000	3.403731000	-0.836759000
H	4.967713000	4.230166000	-1.552288000
H	4.711609000	4.253276000	0.231385000
H	6.098155000	0.051123000	-0.219724000
H	7.364454000	4.422986000	0.191911000

Experimental section

Unless otherwise stated, all glassware was flame-dried before use and all reactions were performed under an atmosphere of argon. All reagents were used as received from commercial suppliers unless otherwise stated. Solvents were purchased as anhydrous over molecular sieves and were used as received. Reaction progress was monitored by thin layer chromatography (TLC) performed on aluminum plates coated with silica gel F254 with 0.2 mm thickness. Chromatograms were visualized by fluorescence quenching with UV light at 254 nm or by staining using potassium permanganate. Flash column chromatography was performed using silica gel 60 (230-400 mesh, Merck and co.). Neat infrared spectra were recorded using a Perkin-Elmer Spectrum 100 FT-IR spectrometer. Wavenumbers (ν_{max}) are reported in cm^{-1}. Mass spectra were obtained using a Bruker maXis UHR-TOF (Qq-TOF) spectrometer, using electrospray ionization (ESI). All ^{1}H NMR and ^{13}C NMR spectra were recorded using a Bruker AV-400 or AV-600 spectrometer at 300K. Chemical shifts are given in parts per million (ppm, δ), referenced to the solvent peak of CDCl3, defined at δ = 7.26 ppm (^{1}H-NMR) and δ = 77.16 (^{13}C-NMR) or DMSO-d6, defined at δ = 2.50 ppm (^{1}H-NMR) and δ = 39.52 (^{13}C-NMR). Coupling constants are quoted in Hz (J). ^{1}H NMR splitting patterns are designated as singlet (s), doublet (d), triplet (t), quartet (q) as they appeared in the spectrum. If the appearance of a signal differs from the expected splitting pattern, the observed pattern is designated as apparent (app). Splitting patterns that could not be interpreted or easily visualized are designated as multiplet (m) or broad (b).

Quench studies using ^{18}O-labeld water

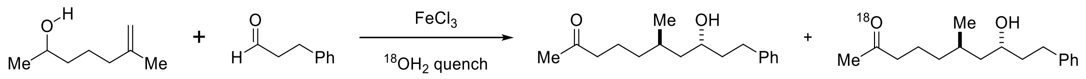

1a **7** **8** **8'**

47%
Ratio **8**:**8'** = 1:5

Alcohol **1a** (0.2 mmol, 25.6 mg, 1.0 eq.) was weighted into a vial, diluted with 2 mL 1,2-dichloroethane and brought to the reaction temperature using an oil bath. Aldehyde (0.24 mmol, 33.9 mg, 1.2 eq.) and $FeCl_3$ (0.01 mmol, 5 mol%) were dissolved in a minimum amount of 1,2-dichloroethane, then added to the reaction mixture. The reaction was heated for 10 minutes at 100 °C. ^{18}O-Water (0.2 mL) was added and the reaction was allowed to cool to room temperature with stirring. The reaction mixture was filtered over a short pad of silica, eluted with dichloromethane and the solvent was removed under reduced pressure to obtain the crude product. Purification by flash column chromatography afforded the pure product (0.09 mmol, 24.9 mg, 47%, 5:1 ratio ^{18}O/O (**8'**/**8**)).

The spectroscopic data of **8** matched the reported data.

^{1}H-NMR (400 MHz, $CDCl_3$): δ 7.37 – 7.29 (m, 2H), 7.25 (d, *J* = 7.6 Hz, 3H), 3.92 – 3.60 (m, 1H), 2.84 (dt, *J* = 15.4, 8.0 Hz, 1H), 2.72 (dt, *J* = 13.9, 7.9 Hz, 1H), 2.45 (t, *J* = 7.3 Hz, 2H), 2.18 (s, 3H), 1.89 – 1.77 (m, 2H), 1.60 (dddt, *J* = 43.8, 17.9, 9.6, 4.6 Hz, 4H), 1.43 (s, 1H), 1.37 – 1.12 (m, 3H), 0.94 (d, *J* = 6.6 Hz, 3H) ppm.

^{13}C-NMR (100 MHz, $CDCl_3$): δ 209.3, 142.28, 128.5 (4C), 125.9, 69.2, 44.9, 44.0, 40.2, 37.3, 32.3, 30.0, 29.2, 21.2, 19.3 ppm.

HRMS (ESI$^+$): exact mass calculated for $[M+H]^+$ ($C_{17}H_{27}O^{18}O$) required *m/z* 265.2048, found *m/z* 265.2056.

FT-IR (neat) n_{max}: 2926, 2868.26 1713, 1678, 1495, 1454, 1408, 1360, 1312, 1268, 1250, 1231, 1163, 1116, 1086, 1052, 1031, 991, 920, 864, 746 cm^{-1}.

NMR Spectra

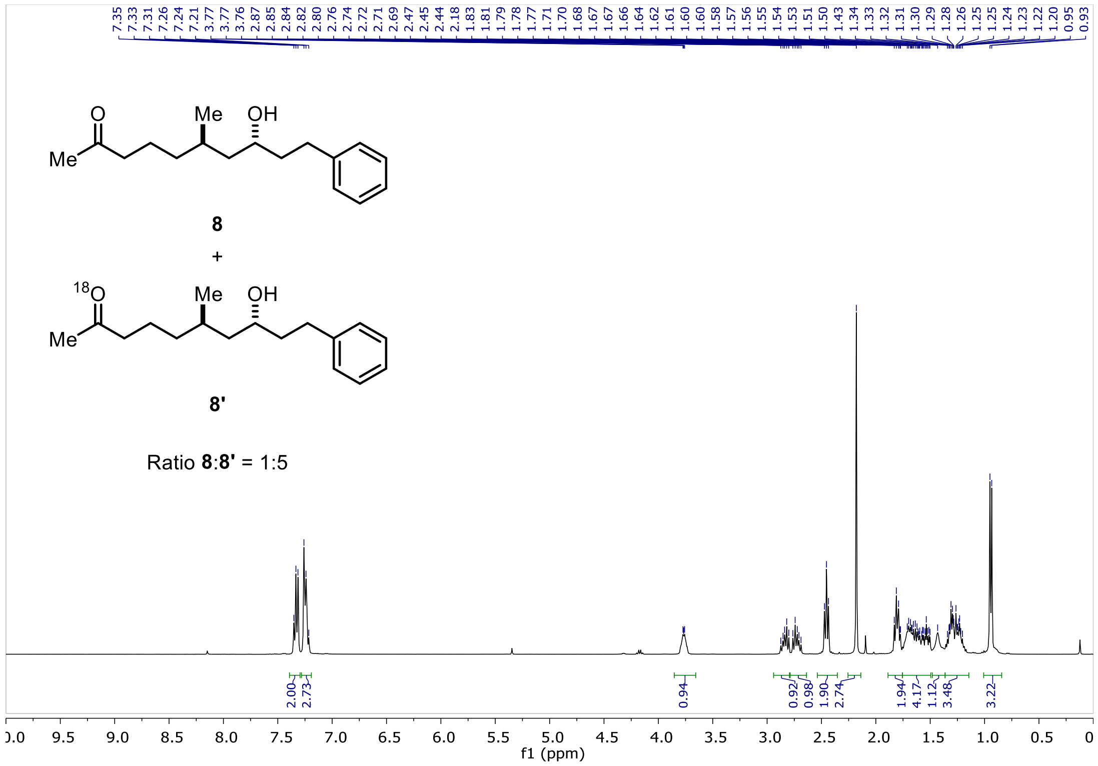

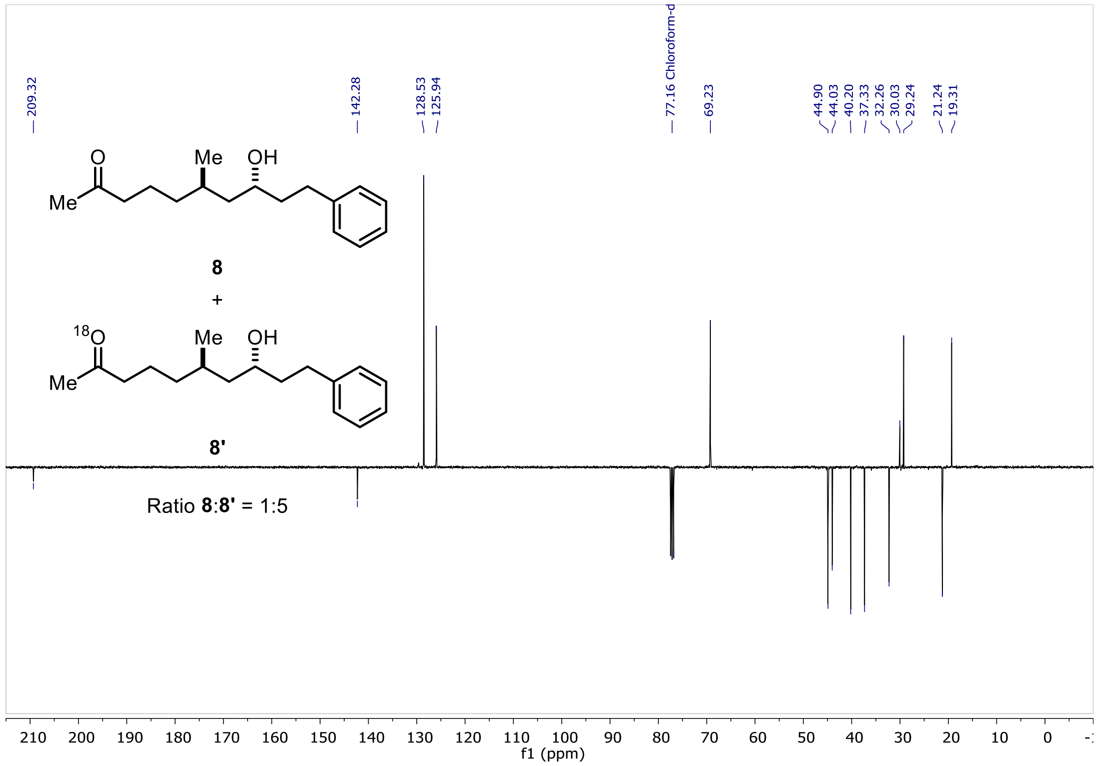

^{13}C-NMR comparison of **8** and **8’**

An authentic sample of compound **8** was synthesized according to a literature procedure and compared to product **8’**.

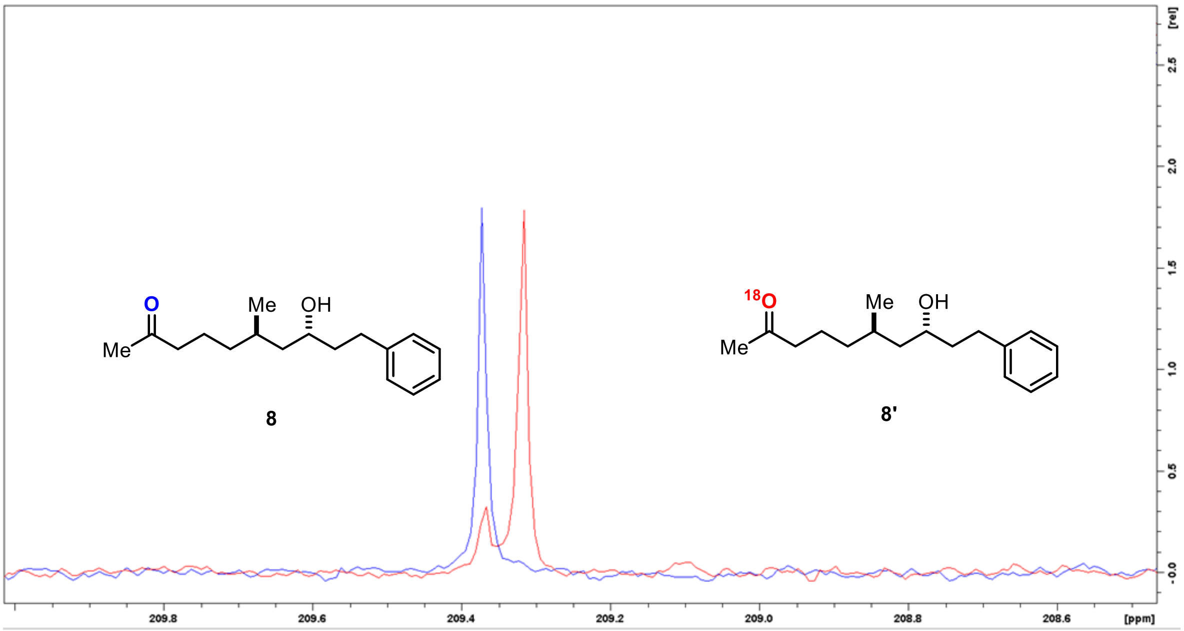

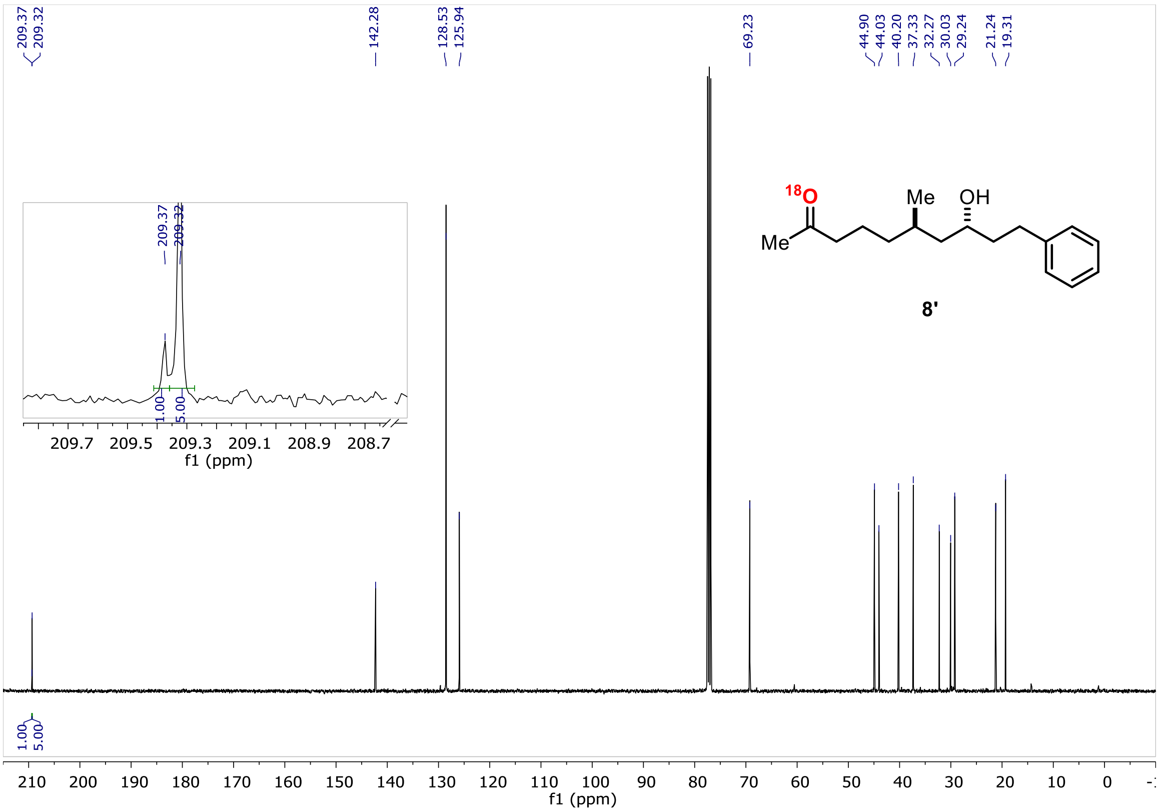

Supplement: Supplementary file 1 — Supporting Information [file CPLU-90-e202400751-s001.pdf]
